# β‐aminobutyric acid does not induce defenses or increase Norway spruce resistance to the bluestain fungus *Grosmannia penicillata*

**DOI:** 10.1111/ppl.70009

**Published:** 2024-12-14

**Authors:** Ngan Bao Huynh, Paal Krokene, Line Nybakken, Vytautas Čėsna, Melissa H. Mageroy

**Affiliations:** ^1^ Division of Biotechnology and Plant Health Norwegian Institute of Bioeconomy Research Ås Norway; ^2^ Faculty of Environmental Sciences and Natural Resource Management, Norwegian University of Life Sciences Ås Norway; ^3^ Institute of Forestry, Lithuanian Research Centre for Agriculture and Forestry Girionys Lithuania

## Abstract

Priming of Norway spruce (*Picea abies*) inducible defenses is a promising way to protect young trees from herbivores and pathogens. Methyl jasmonate (MeJA) application is known to induce and potentially prime Norway spruce defenses but may also reduce plant growth. Therefore, we tested β‐aminobutyric acid (BABA) as an alternative priming chemical to enhance spruce resistance, using 2‐year‐old Norway spruce plants. We compared inducible defense responses, i.e. traumatic resin duct formation and accumulation of defensive metabolites, in bark and xylem tissues of BABA‐ or MeJA‐treated plants before and after wounding. We also evaluated the effect of these chemical treatments on Norway spruce resistance to the pathogenic bluestain fungus *Grosmania penicilliata*. BABA did not induce defense responses or pathogen resistance, it even reduced concentrations of total terpenes in the treated plants. In contrast, MeJA induced traumatic resin duct formation, accumulation of flavonoids, pathogen resistance, and did not affect plant growth. For the first time, flavan‐3‐ols (catechins) were shown to have a primed response to MeJA treatment in Norway spruce. Our results indicated that BABA is not a suitable alternative priming chemical to MeJA in Norway spruce.

## 1 INTRODUCTION

Norway spruce (*Picea abies* L. H. Karst) is an ecologically and economically important tree species in boreal and subalpine forests, from Central and Northern Europe in the west to the Ural Mountains in the east (Hannrup et al., 2004, Caudullo et al., 2016). Across its wide geographical range, Norway spruce must protect itself against multiple abiotic and biotic stresses. A particularly destructive biotic stressor is the tree‐killing bark beetle *Ips typographus* and beetle‐associated fungal pathogens, such as *Grosmannia penicillata* (Krokene, 2015, Zhao et al., 2019).

To better protect spruce trees from bark beetles and pathogens it is important to understand tree defenses and, if possible, develop methods to increase tree resistance. Norway spruce trees have complex defenses that include mechanical and chemical defenses (Christiansen et al., 1987, Lieutier and Battisti, 2004, Krokene, 2015). Mechanical defenses are structural components, such as lignin or suberin polymers, that reinforce tissues and act as barriers to invaders (Whitehill et al., 2023). Chemical defenses consist primarily of secondary metabolites with toxic or inhibitory effects (Krokene, 2015). One important group of defense chemicals in conifers are terpenes, which function as a defense against bark beetles, their fungal symbionts, and other pests and pathogens (Krokene, 2015). Even though they are costly to synthesize, terpenes are produced in large quantities (Keeling and Bohlmann, 2006, Krokene, 2015). Terpenes make up most of the trees' viscous and sticky resin, which can trap attackers and seal wounds (Krokene, 2015). Phenolics are also important conifer defense chemicals and are considered to have antifungal properties and inhibit insect feeding, among other functions (Chong et al., 2009, Faccoli and Schlyter, 2007, Lieutier and Battisti, 2004). In conifers, phenolic compounds are synthesized, modified, and stored in specialized cells called polyphenolic parenchyma (PP) cells, as well as in other cell types (Franceschi et al., 1998, Franceschi et al., 2000).

Conifer defenses can be constitutively present or induced. Constitutive defenses, such as cork bark and terpenoid resin, provide baseline resistance against attackers (Krokene, 2015). Inducible defenses are activated in response to an external stimulus, such as beetle attack, and provide a second line of defense. Inducible defenses can be categorized as direct, prolonged, or primed, based on when they are activated relative to the external stimulus (Wilkinson et al., 2019). Direct induction is activated immediately after the stimulus and includes the formation of traumatic resin ducts (TRDs) and the activation of existing axial resin ducts (Nagy et al., 2000) and PP cells (Franceschi et al., 1998, Krokene, 2015). Most directly induced defense responses are completed or return to constitutive levels after a few weeks, but some are maintained for longer (Mageroy et al., 2020b) and are classified as prolonged induced defenses (Wilkinson et al., 2019). Prolonged upregulation of defenses is costly, as defenses are continuously maintained after activation (Mauch‐Mani et al., 2017, Wilkinson et al., 2019). A more cost‐effective strategy is the priming of defenses, where, initially, plant defenses are only mildly and transiently induced by exposure to a priming stimulus, which can be mechanical damage, failed insect attack, or chemical application. However, the priming stimulus also sensitizes plant defenses and enables a faster and stronger response to a later triggering stimulus, such as an insect attack or fungal infection (Martinez‐Medina et al., 2016, Mauch‐Mani et al., 2017).

Long‐term induced resistance in Norway spruce was first demonstrated by Christiansen and co‐authors, who wounded or applied sub‐lethal fungal infections on trees before challenging them with a massive fungal infection 13–15 days after initial wounding/inoculation. Pre‐treated trees turned out to be much more resistant to the massive fungal infection than naive trees (Christiansen et al., 1999). Since this initial study, many studies have shown that long‐term induced resistance in Norway spruce can be stimulated by mechanical wounding, sub‐lethal fungal inoculations, or treatment with chemical elicitors such methyl jasmonate (MeJA), a derivative of jasmonic acid (Martin et al., 2002, Erbilgin et al., 2006, Krokene et al., 2008, Zeneli et al., 2006). More recently, MeJA‐induced resistance in Norway spruce has been shown to result from a combination of prolonged induced and primed defense responses (Mageroy et al., 2020a, Mageroy et al., 2020b, Wilkinson et al., 2022).

Despite its effectiveness as an elicitor of long‐term induced resistance in spruce, MeJA treatment also has some negative effects (Huynh et al., 2024). Several studies have demonstrated that MeJA application reduces height growth in Norway spruce and other conifers (Gould et al., 2009, Heijari et al., 2005a, Moreira et al., 2012b, Sampedro et al., 2011, Vivas et al., 2012, Zas et al., 2014). Other negative effects of MeJA application include reduced tracheid cell lumen area, reduced net photosynthetic rate and stomatal closure (Heijari et al., 2005a), and reduced sapwood growth (Krokene et al., 2008, Krokene et al., 2023). Furthermore, the high cost of MeJA limits its practical use as a plant protection tool (Huynh et al., 2024). Due to these negative aspects of MeJA, it is of interest to identify alternative defense priming chemicals for Norway spruce.

A possible alternative to MeJA is β‐aminobutyric acid (BABA). BABA has a well‐documented priming effect in angiosperm crop and model plants, such as Arabidopsis (*Arabidopsis thaliana*), tobacco (*Nicotiana tabacum*), tomato (*Solanum lycopersicum*), and potato *Solanum tuberosum*; (Hönig et al., 2023). BABA‐induced resistance is not regulated through a single defense hormone signaling pathway, but the activated resistance mechanism varies depending on the nature of the triggering stimulus (Cohen et al., 2016). Because BABA has not yet been tested in conifers, we wanted to determine if BABA can induce and/or prime defenses in Norway spruce. Our main objectives were: (1) to compare defense responses induced by MeJA and BABA in Norway spruce plants, and (2) to evaluate if BABA could be an alternative chemical priming stimulus for Norway spruce.

## 2 MATERIALS AND METHODS

**FIGURE 1:**
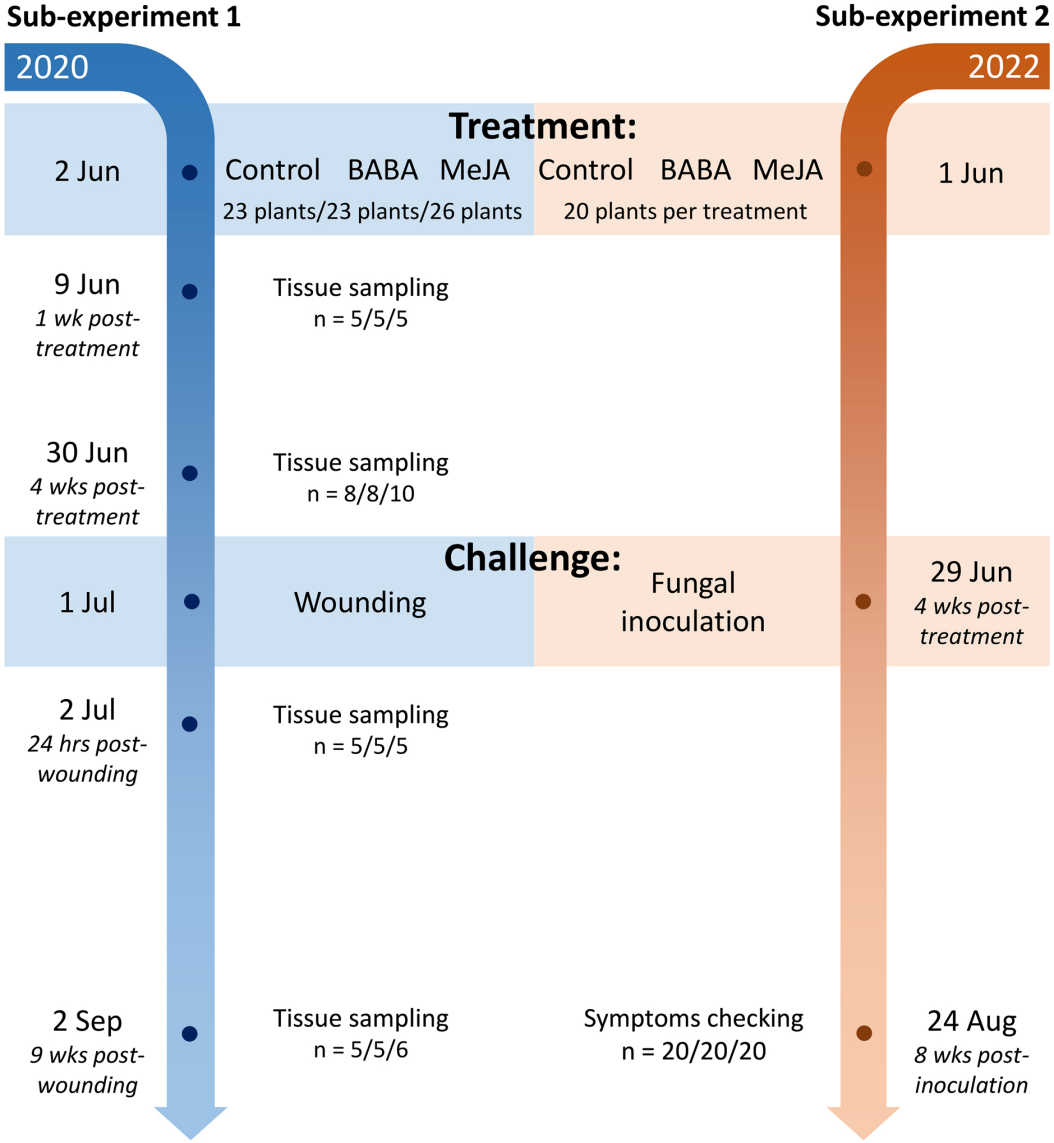
Experimental design of sub‐experiment 1 and 2. Norway spruce plants were first treated with the priming chemicals BABA and MeJA, then challenged with wounding (sub‐experiment 1) or fungal inoculation (sub‐experiment 2) four weeks later. In sub‐experiment 1, tissues were sampled for analysis of defense chemicals one and four weeks after priming treatments and 24 hours and nine weeks after wounding. Both sub‐experiments ended 8–9 weeks after wounding or fungal inoculation. BABA: β‐aminobutyric acid; MeJA: methyl jasmonate.).

Our two main objectives were addressed in two separate sub‐experiments (Figure 1). Briefly, we applied BABA or MeJA to Norway spruce plants and challenged the plants with wounding or fungal infection four weeks later. In sub‐experiment 1, we compared levels of defense chemicals in BABA‐ and MeJA‐treated plants before and after wounding of the stem (objective 1). In sub‐experiment 2, we compared the resistance of treated plants to fungal infection (objective 2).

### 2.1 Sub‐experiment 1

Defense responses of MeJA‐ and BABA‐treated Norway spruce plants.

#### 2.1.1 Plant material, chemical treatments, and tissue sampling

On April 28, 2020, 2‐year‐old Norway spruce plants (1114 Sanderud seed origin; www.skogfroverket.no) obtained from a commercial nursery (Skogplanter Østnorge AS; www.skogplanter.no) were planted in a commercial peat‐based growth medium (Plantejord, TJERBO, Torv & jordprodukter) in individual 0.8‐liter pots (7.5 cm × 7.5 cm × 12 cm; Nelson Garden, Product no. 5726). Plants were kept in a growth room under a photoperiod of 20 hours of light and 4 hours of darkness (light source: Osram L36W/77; 1400 lumens; FLUORA; 100 μmol m^−2^ s^−1^ photosynthetically active radiation (PAR)). The room temperature ranged between 20 and 22°C.

On June 2, 2020, the plants were treated with 50 mM MeJA (26 plants) or 50 mM BABA (23 plants) dissolved in 0.1% Tween and distilled water (MeJA: #392707; BABA: #A44207‐5G; www.sigmaaldrich.com), or with a control solution (Tween and distilled water; 23 plants; Figure 1). MeJA, BABA, and control solutions were applied exogenously by painting the stem surface with a small brush from the bottom of the apical shoot to the soil line (Supplementary Figure S1). Tissue samples for quantification of defense chemicals were destructively harvested one week after treatment (*n* = 5 plants per treatment) and four weeks after treatment (*n* = 8 plants for control and BABA; *n* = 10 for MeJA; Figure 1). All remaining plants were wounded on July 1, 2020, to induce defense responses. A bark flap was cut with a scalpel on the upper stem, just below the first branch whorl, exposing the xylem surface. The bark flap was then immediately closed and wrapped with parafilm. Tissue samples for quantification of defense chemical were again harvested destructively 24 hours after wounding (n = 5 plants per treatment) and nine weeks after wounding (n = 5 plants for control and BABA; *n* = 6 for MeJA; Figure 1). Plant height growth was measured at three time points: before chemical treatment, at the time of wounding four weeks after chemical treatment, and at the end of the experiment nine weeks after wounding (Supplementary Figure S1).

During the tissue sampling, the stem of the harvested plants was cut at the soil line and 10 cm above, lateral branches were removed, and the bark was separated from the xylem. For microscopy, a 4‐cm section of the lower part of the stem was removed before separating the bark and xylem (Supplementary Figure S1). All samples were flash‐frozen in liquid nitrogen and stored at −80°C. Bark tissues were ground to a fine powder with liquid nitrogen using a mortar and pestle and were used to analyze terpenes and phenolics. Xylem tissues were used for terpene analysis only, since phenolic levels in Norway spruce xylem are very low (Erbilgin et al., 2006), and were cut into 1 cm pieces for further processing.

#### 2.1.2 Phenolics analysis

Phenolic extraction and analysis protocols were based on Nybakken et al. (2021). Ground bark samples were freeze‐dried for two days using a LyoQuest‐55 freeze‐dryer (Azbil Telstar Technologies S.L.U.) and stored at −20°C. Samples were thawed for 24 hours before further processing. For each sample, 10 mg ± 2 mg bark powder was weighed out. Samples were transferred to Precellys vials (BERTIN Technologies), together with two zirconium oxide balls and 400 μL methanol (MeOH), and the mixture was homogenized for 30 seconds using a Precellys 24 tissue homogenizer (BERTIN Technologies) at 10 500 rcf. Samples were then placed in an ice bath for 15 minutes, centrifuged for 3 minutes at 25 000 rcf in an Eppendorf 5417C centrifuge (Eppendorf AG), and the supernatant was transferred to a 5 mL collecting glass tube. This process was repeated three times for each sample, or until the solid sample residue was colorless. The remaining MeOH in the collecting glass tube was evaporated using an Eppendorf Concentrator Plus vacuum centrifuge (Eppendorf AG) and the glass tubes were stored at −20°C before putting them into an HPLC system. The remaining solid residues were kept in the Precellys vial and stored at −20°C until further analysis for MeOH‐insoluble tannins.

After the collecting glass tubes were thawed for 20 minutes, 200 μL MeOH and 200 μL pure H_2_O from a Purelab CHORUS 1 water purifier (ELGA Labwater, Veolia Water Technologies) was added. Samples were dissolved by immersing the tubes in an Ultrasonic Cleaners water bath (USC200TH, VWR), transferred to Eppendorf tubes, centrifuged for 3 minutes at 25 000 rcf to remove residues, and then transferred to HPLC vials. Low‐molecular‐weight phenolic compounds were analyzed using an Agilent Technologies High‐Throughput Liquid Chromatography (HPLC) system (Agilent Technologies 1100 series) with the associated Agilent ChemStation software for the LC 3D systems. The Agilent system consisted of a G1312A binary pump with a G1322A degasser, a G1330A thermostat module, a G1329A autosampler, a G1315 diode array detector, and a G1316A column compartment. A 50 mm × 4.6 mm HPLC column (ODS HYPERSIL 3 μm, reversed‐phased C18, octadesyl‐silica, Thermo Fisher Scientific) was used. Samples were eluted with a flow rate of 2 mL min^−1^ using a gradient from solution A (30 mL tetrahydrofuran, 5 mL orthophosphoric acid (85%) and pure water for a total volume of 2000 mL) to solution B [HPLC gradient grade MeOH (VWR Chemical)]. The gradient is given in Table S7. The injection volume was 20 μL and the temperature was 30°C during the whole analysis. Identifications and quantifications of low‐molecular weight phenolic compounds were achieved by comparing retention times and absorption spectra at 270 and 320 nm with those of commercial standards.

MeOH‐soluble tannins were analyzed from the remaining samples after HPLC analysis, whereas MeOH‐insoluble tannins were analyzed from the solid residues after the extraction of soluble phenolics. Each sample was divided into two and transferred to two glass tubes. For MeOH‐soluble tannins, each tube contained 100 μL sample, or 50 μL sample if there was not enough remaining liquid in the HPLC vial. For MeOH‐insoluble tannins, each tube contained a minimum of 1 mg and a maximum of 3 mg of solid residues. We then added 400 μL MeOH (or 450 μL for 50 μL samples), 100 μL ferric reagent, and 3 mL butanolic acid to each tube. The tubes were closed with a cap, boiled in a water bath (VWB2 26) at 99°C for 50 minutes, and then cooled to room temperature. The liquid in each tube was transferred to a 4.5 mL VWR Cuvettes PS Macro. Absorbance at 550 nm was determined using a UV spectrophotometer (UV‐1800, Shimadzu Corporation) equipped with UVProbe 2.62 software. Formulas for calculating concentrations of MeOH‐insoluble and ‐soluble tannins are given in the supplementary information.

#### 2.1.3 Terpene analysis

For terpene analysis, 10 mg of ground bark tissues or a 1 cm segment of xylem tissues was soaked in 1 mL hexane (containing 10 μg ml^−1^ pentadecane as an internal standard) for 24 hours with shaking. The supernatant was transferred to a new vial and stored at −20°C until analysis. The remaining bark or xylem tissue was dried in a fume hood to determine sample dry weight. Terpene extracts were analyzed using a Gas Chromatography–Mass Spectrometry (GC–MS) system with a Varian 3400 gas chromatograph (Hewlett Packard) equipped with a DB‐wax capillary column of 30 m × 0.25 mm × 0.25 mm (J&W Scientific) and connected to a Finnigan SSQ 7000 mass spectrometer. Individual terpenes were identified and quantified by searching for components in the National Institute of Standards and Technology (NIST) database using the AMDIS software. Due to the large number of terpenes present in Norway spruce, only a few major terpenes with known biological functions were quantified. Relative terpene concentrations were calculated by normalizing chromatogram peak areas to that of the internal standard and the sample dry weight. The formula used to calculate terpene concentrations is given in the supplementary information.

#### 2.1.4 Microscopy analysis of traumatic resin ducts

Formation of traumatic resin ducts (TRDs) in the xylem was evaluated using a Leica Microsystems microscope (Wetzlar GmbH), equipped with Leica Application Suite software (version 4.13.0). Thin and level stem cross‐sections were cut by hand about 6 cm above the soil line using a platinum‐coated razor blade (Feather). Stem cross‐sections were examined under three magnifications: 2.5× was used to measure the total xylem area per section and to observe if TRDs were present; 5× and 10× were used for closer inspection of TRDs and measurement of TRD lumen area. The formula used to calculate TRD coverage per cross‐sectional xylem area is given in the supplementary information.

### 2.2 Sub‐experiment 2

Resistance of MeJA‐ and BABA‐treated Norway spruce plants to *Grosmannia penicillata*.

#### 2.2.1 Plant material and chemical treatments

On October 7, 2021, 2‐year‐old Norway spruce plants (1116 Romedal; www.skogfroverket.no) obtained from a commercial nursery (Skogplanter Østnorge AS; www.skogplanter.no) were planted and grown under the same conditions as in sub‐experiment 1. On June 1, 2022, the plants were treated with 1 mM MeJA or BABA dissolved in 0.1% Tween and distilled water or with a control solution consisting of Tween and distilled water. Chemical treatments were applied by soil drenching, as stem application of BABA in sub‐experiment 1 did not induce any defense responses (see Results section below). Each plant received 10 mL solution, with 5 mL pipetted into the soil at two different sites 5 cm away from the stem base.

#### 2.2.2 Inoculation with *Grosmannia penicillata*

*Grosmannia penicillata* isolate 1980–91/54 (collected in 1980 at Slørstad, Ås, Akerhus, Norway) from the culture collection of the Norwegian Institute of Bioeconomy Research was taken out from a − 150°C stock culture and plated on malt agar (1.25% malt, 2% agar). Approximately four weeks before inoculation, fresh malt agar plates were inoculated with fungus. On June 29, 2022, five plants from each treatment were inoculated with either sterile malt agar or malt agar colonized by *G. penicillata*. The sterile or fungal inoculum was homogenized by squeezing the agar back and forth several times between two 60 mL syringes. To increase the inoculation load, each plant was inoculated at two closely spaced locations on the upper stem (Supplementary Figure S1). A 5 mm cork borer was used to cut a bark flap at the inoculation site and inoculum was placed inside the wound using a 5 mL needle‐free syringe. The bark flap was then closed, and the site was wrapped with parafilm. Eight weeks after inoculation, on August 24, 2022, the bark at each inoculation site was peeled off to expose the xylem, and fungal infection was assessed by checking for the presence of necrotic lesions on the xylem surface. If there was black discoloration at the wound site the plant was classified as “infected”, if there was no discoloration it was classified as “not infected” (Supplementary Figure S2).

### 2.3 Statistical analysis

All statistical analyses were performed in RStudio (version 4.1.2). Two‐way ANOVA models with interaction were used to assess the effects of chemical treatment and time since treatment or wounding on chemical defense responses (phenolic and terpene concentrations), using the function “lm” (from the “stats” package). ANOVA tables for the models were obtained using the function “Anova” from the “mixlm” package (Liland and Sæbø, 2014). All models were checked visually for assumptions of normality, constant variance, and independence using the function “plot”. All models were then subjected to Tukey's pairwise post‐hoc test using the function “emmeans” from the package “emmeans” (Lenth, 2023). Pairwise comparisons were made to compare different chemical treatments within time points as well as different time points for each treatment. Amounts of TRDs for different chemical treatments were assessed using one‐way ANOVA, followed by a Tukey's pairwise post‐hoc test, using the same procedure and functions as above. Effects of chemical treatments on lesion incidence were assessed by Fisher's exact test using the function “fisher.test”, followed by post‐hoc pairwise comparison using the function “pairwise_fisher_test” from the package “rstatix” (Kassambara, 2023). Bar plots were made using the package “ggplot2” (Wickham, 2016) and “patchwork” (Pedersen, 2022).

## 3 RESULTS

### 3.1 Defense responses of MeJA‐ and BABA‐treated Norway spruce

In sub‐experiment 1, we investigated chemical and anatomical defense responses induced by BABA and MeJA in 2‐year‐old Norway spruce plants. We quantified TRD formation and induction of terpenes and phenolics at two time points after BABA/MeJA treatment and two time points after stem wounding.

#### 3.1.1 Traumatic resin ducts

**FIGURE 2:**
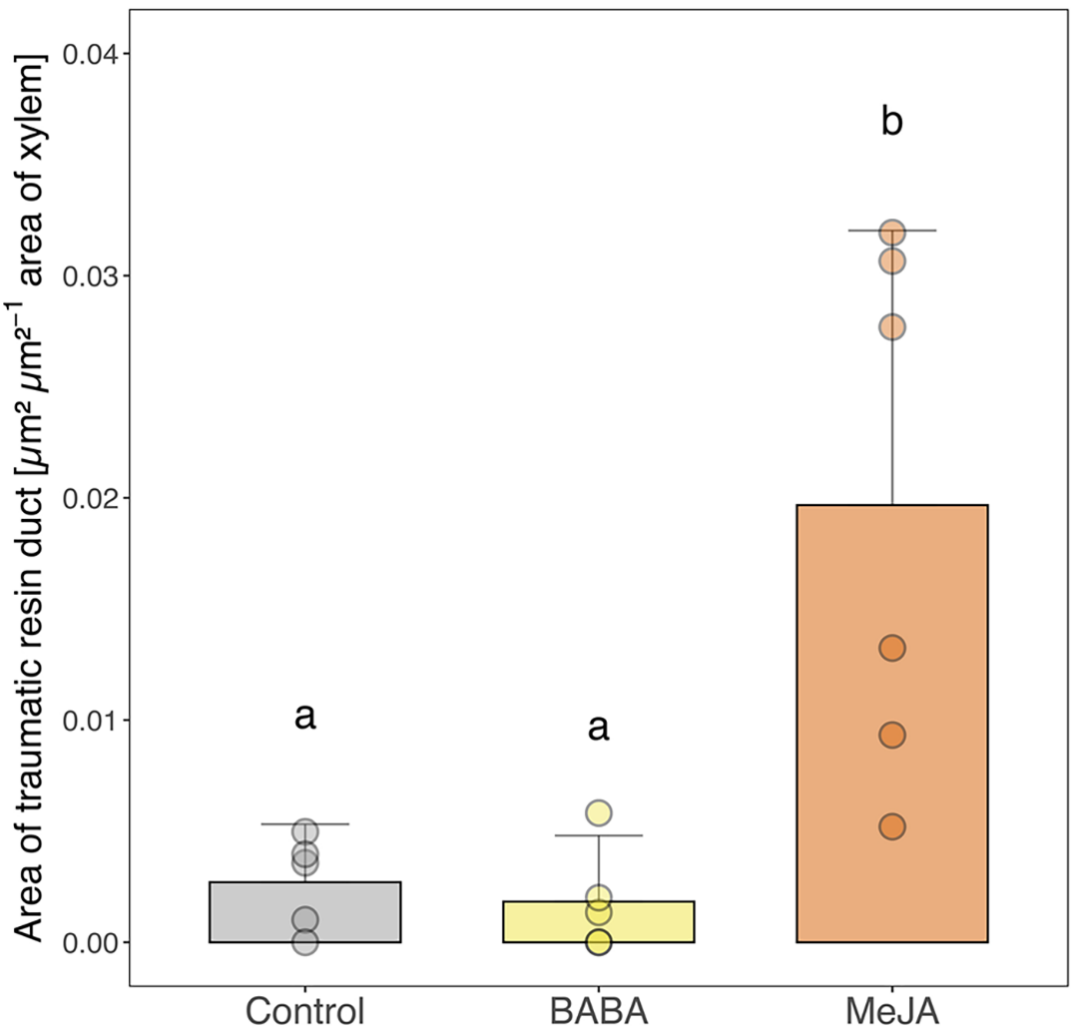
Traumatic resin duct formation. Mean area of traumatic resin ducts per cross‐sectional area of xylem in Norway spruce plants 13 weeks after treatment with water and Tween (Control), β‐aminobutyric acid (BABA) or methyl jasmonate (MeJA) and nine weeks after wounding of the stem. Error bars represent 95% confidence intervals and circles represent individual data points (*n* = 5 per treatment, except for MeJA where *n* = 6). Treatments with different letters are significantly different (1‐way ANOVA followed by Tukey's HSD post hoc test, *p* < 0.05).

Because exploratory analyses showed that no TRDs were visible in any samples until 9 weeks after wounding, we only analyzed 9‐week‐samples for TRDs. Microscopy analysis of TRD formation showed that plants treated with MeJA had significantly more TRDs than control plants and plants treated with BABA (Figure 2).

#### 3.1.2 Terpenes

**FIGURE 3:**
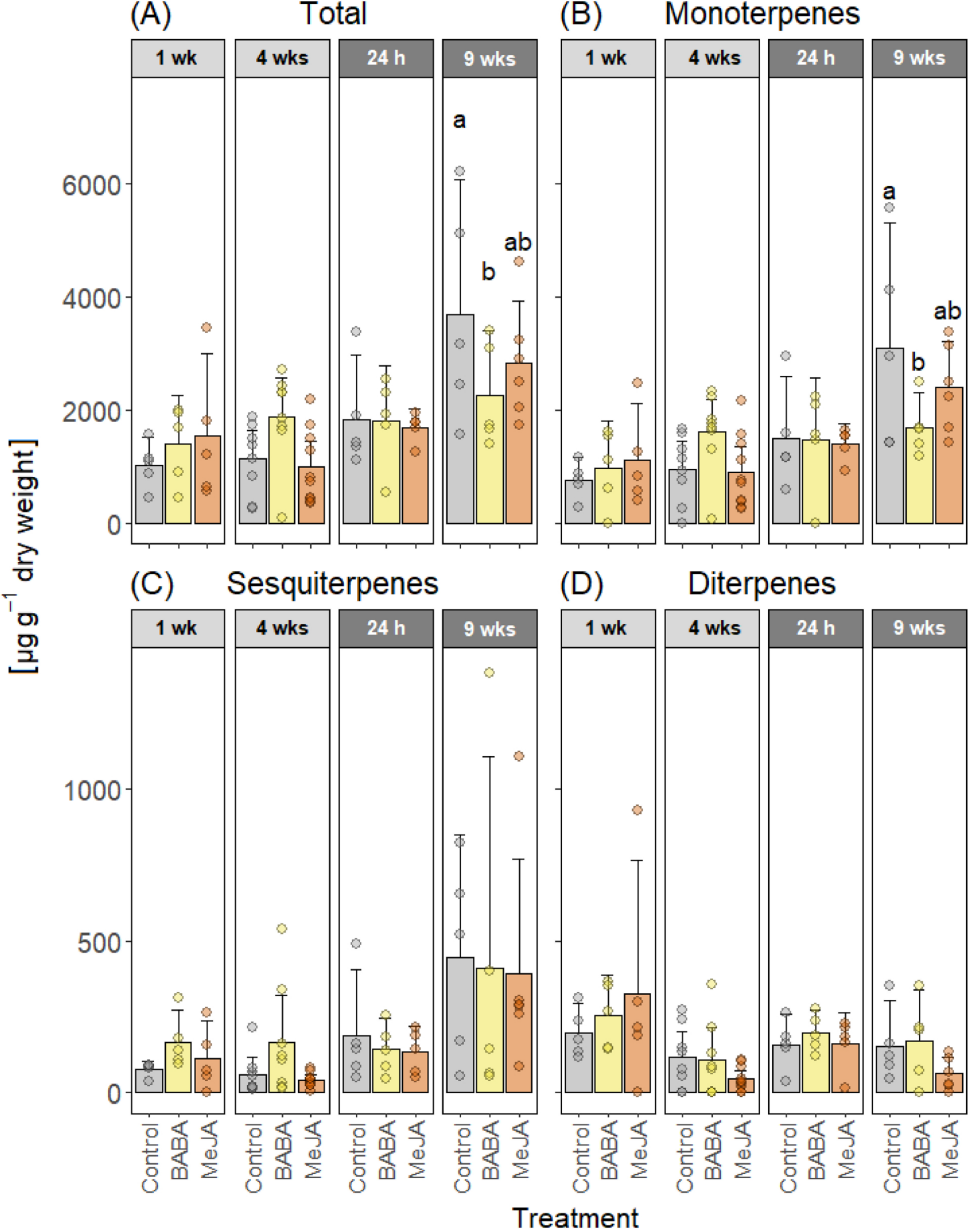
Terpene concentrations in the bark. Mean concentrations of (A) total terpenes, (B) monoterpenes, (C) sesquiterpenes, and (D) diterpenes in Norway spruce bark tissues after treatment with water and Tween (Control), β‐aminobutyric acid (BABA), or methyl jasmonate (MeJA). The bark was wounded four weeks after treatment. Terpene concentrations were measured oneweek (1 wk) and four weeks (4 wks) after treatment (light grey panels) and 24 hours (24 h) and nine weeks (9 wks) after wounding (dark grey panels). Error bars show 95% confidence intervals and circles represent individual data points (n = 5 to 10 plants per treatment and time point, see Figure 1). For each terpene class and time point, treatments with different letters are significantly different (2‐way ANOVA followed by Tukey's HSD post hoc test, p < 0.05).

A total of twelve monoterpenes, three sesquiterpenes, and two diterpenes were identified in bark and xylem tissues using GC–MS. Concentrations and statistical comparisons for individual terpenes in bark and xylem can be found in Supplementary Table S1a, S1b, S2a, and S2b. In the bark, concentrations of total terpenes or total mono‐, sesqui‐ or diterpenes did not differ significantly between control plants and MeJA‐treated plants at any time point. BABA‐treated plants, however, had significantly lower concentrations of total terpenes compared to control trees nine weeks after wounding, and this was driven by monoterpenes (Figure 3, Supplementary Table S1a and S1b).

**FIGURE 4:**
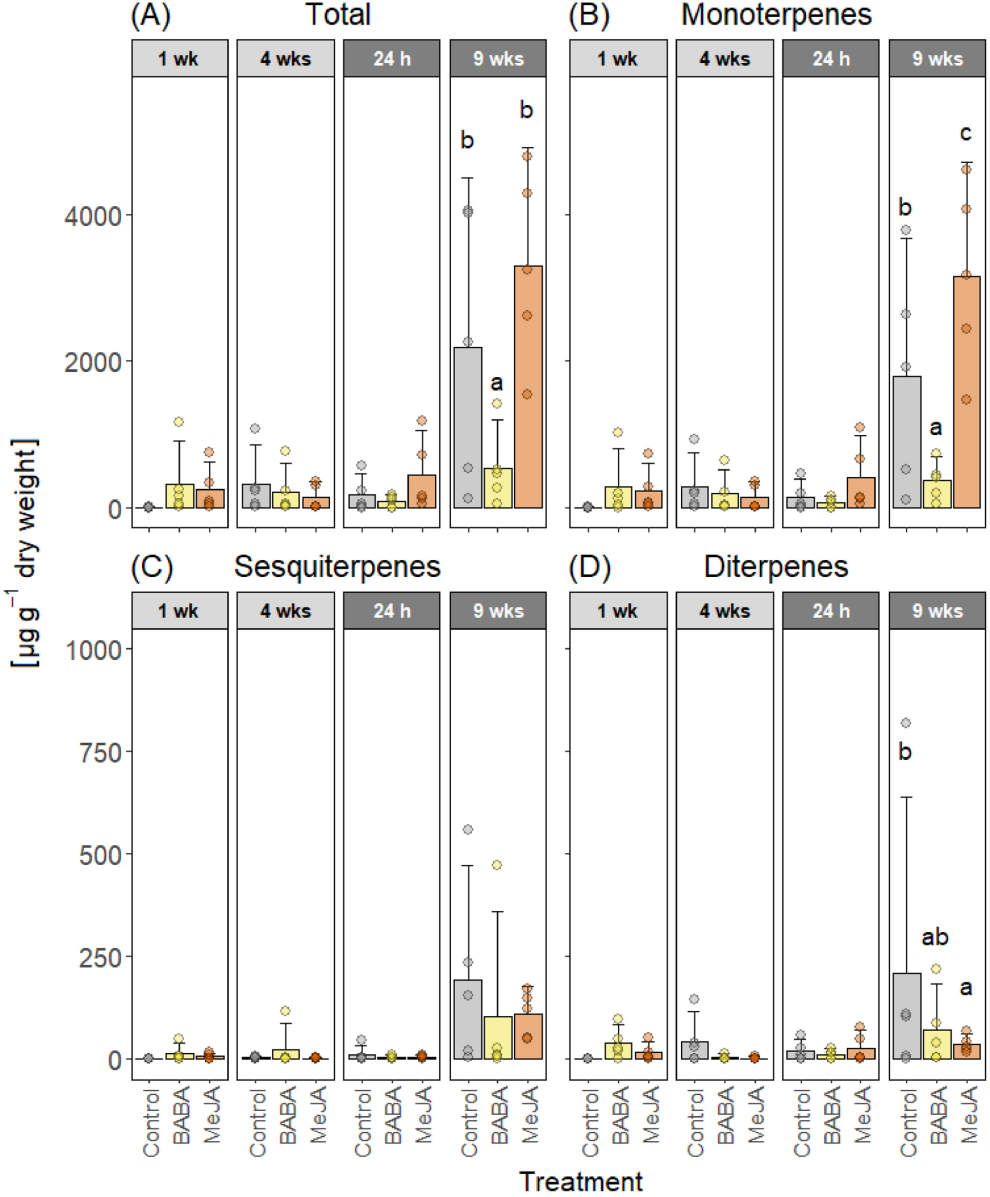
Terpene concentrations in the xylem. Mean concentrations of (A) total terpenes, (B) monoterpenes, (C) sesquiterpenes, and (D) diterpenes in Norway spruce xylem after treatment with water and Tween (Control), β‐aminobutyric acid (BABA), or methyl jasmonate (MeJA). The bark was wounded four weeks after treatment. Terpene concentrations were measured one week (1 wk) and four weeks (4 wks) after treatment (light grey panels) and 24 hours (24 h) and nine weeks (9 wks) after wounding (dark grey panels). Error bars show 95% confidence intervals and circles represent individual data points (n = 5 per treatment and time point). For each terpene class and time point, treatments with different letters are significantly different (2‐way ANOVA followed by Tukey's HSD post hoc test, p < 0.05).

In the xylem, BABA‐treated plants also had significantly lower concentrations of total terpenes and monoterpenes than control plants nine weeks after wounding (Figure 4, Supplementary Table S2a and S2b). Unlike the bark, MeJA‐treated plants had significantly more total monoterpenes in the xylem than control plants nine weeks after wounding. However, MeJA‐treated plants had significantly lower concentrations of diterpenes in the xylem. Thus, total terpene concentrations in the xylem of MeJA‐treated plants did not differ significantly from control plants (Figure 4).

#### 3.1.3 Phenolics

**FIGURE 5:**
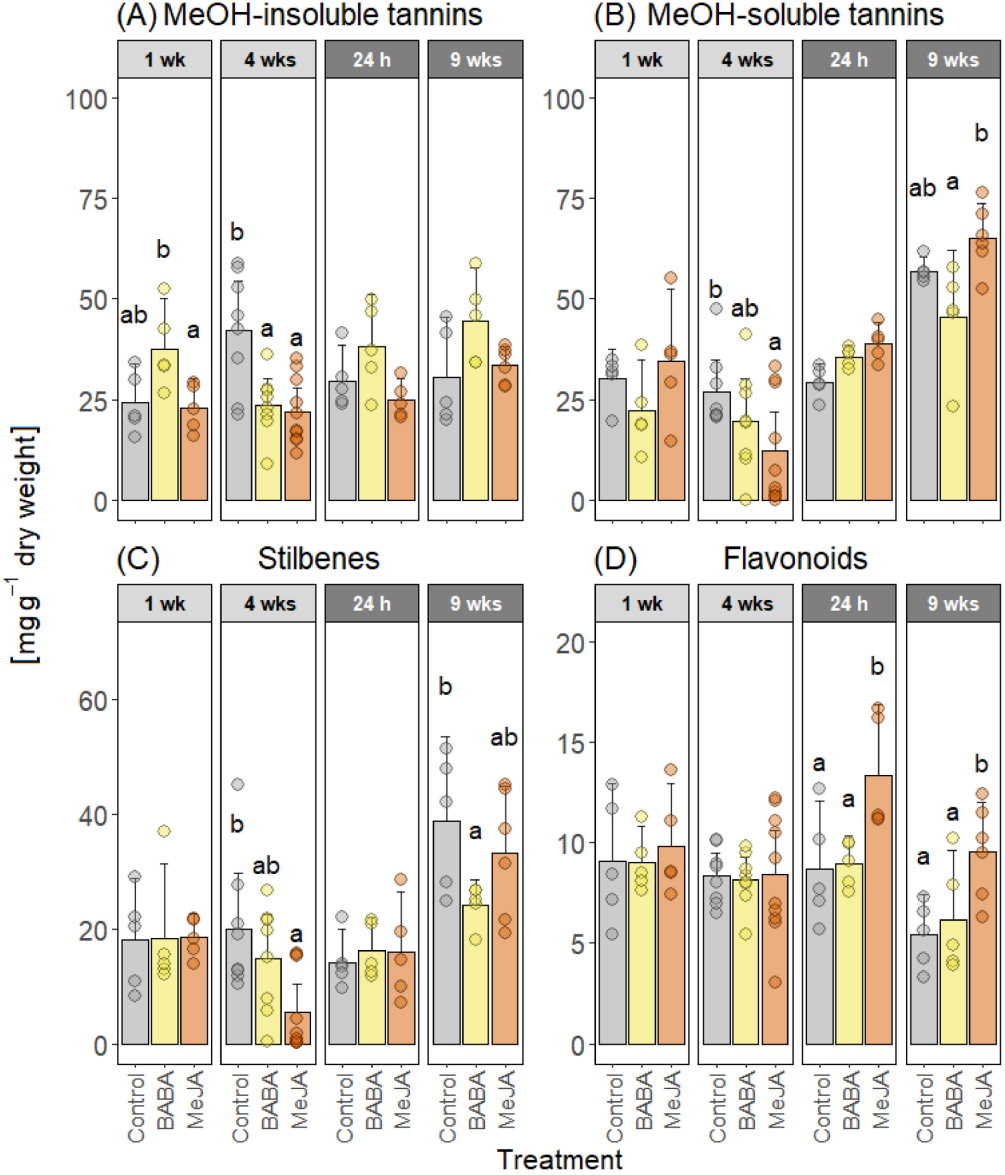
Phenolic concentrations in the bark. Mean concentrations of (A) MeOH‐insoluble tannins, (B) MeOH‐soluble tannins, (C) stilbenes, and (D) flavonoids in Norway spruce bark tissues after treatment with water and Tween (Control), β‐aminobutyric acid (BABA), or methyl jasmonate (MeJA). The bark was wounded four weeks after treatment. Chemical concentrations were measured one week (1 wk) and four weeks (4 wks) after treatment (light grey panels) and 24 hours (24 h) and nine weeks (9 wks) after wounding (dark grey panels). Error bars show 95% confidence intervals and circles represent individual data points (n = 5 to 10 per treatment and time point, see Figure 1). For each compound and time point, treatments with different letters are significantly different (2‐way ANOVA followed by Tukey's HSD post hoc test, p < 0.05).

We also quantified concentrations of phenolic compounds in the bark, including picein, 10 flavonoids, eight stilbenes, and MeOH‐insoluble/−soluble tannins (Figure 5, Supplementary Table S1a and S1b). Concentrations and statistical comparisons for individual phenolic compounds and compound groups can be found in Supplementary Table S3a and S3b.

Four weeks after BABA/MeJA treatment, the concentration of MeOH‐insoluble tannins in both BABA‐ and MeJA‐treated trees was significantly lower than in the control trees (Figure 5). MeOH‐soluble tannins were significantly lower in MeJA‐treated plants relative to control plants four weeks after treatment, but at nine weeks after wounding there was no significant difference between these treatments. MeJA‐treated plants had significantly higher concentration of MeOH‐soluble tannins than BABA‐treated plants nine weeks after wounding. Total stilbene concentration was significantly lower in MeJA‐treated plants than in control plants four weeks after treatment but there were no significant differences after wounding. BABA‐treated plants, on the other hand, had significantly lower stilbene concentrations than control plants nine weeks after wounding. Unlike the other phenolic compounds we analyzed, flavonoid concentrations were significantly higher in MeJA‐treated plants than in control plants and BABA‐treated plants at both time points after wounding.

**FIGURE 6:**
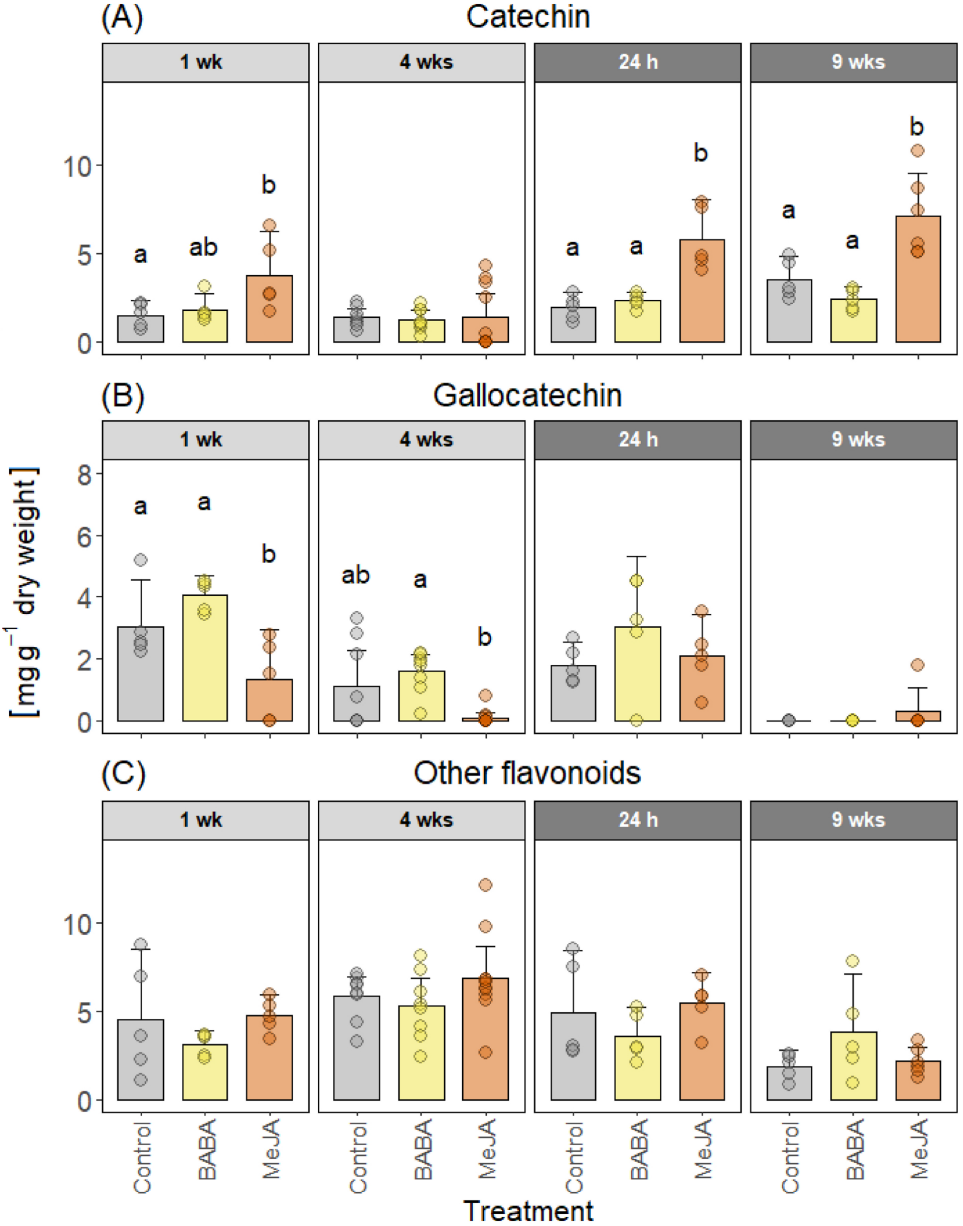
Flavonoid concentrations in the bark. Mean concentrations of flavonoids (A) catechin, (B) gallocatechin, and (C) others in Norway spruce bark tissues after treatment with water and Tween (Control), β‐aminobutyric acid (BABA), or methyl jasmonate (MeJA). The bark was wounded four weeks after treatment. Chemical concentrations were measured one week (1 wk) and four weeks (4 wks) after treatment (light grey panels) and 24 hours (24 h) and nine weeks (9 wks) after wounding (dark grey panels). Error bars show 95% confidence intervals and circles represent individual data points (n = 5 to 10 per treatment and time point, see Figure 1). For each compound and time point, treatments with different letters are significantly different (2‐way ANOVA followed by Tukey's HSD post hoc test, p < 0.05).

The higher concentration of total flavonoids in MeJA‐treated plants was mainly due to a higher level of (+)‐catechin. (+)‐Catechin concentrations were significantly higher in MeJA‐treated plants than control plants one week after treatment, but at four weeks there was no significant differences between the treatments (Figure 6). After wounding, (+)‐catechin concentrations were higher in MeJA‐treated plants than in BABA‐treated and control plants at both time points. Gallocatechin concentrations were significantly lower in MeJA‐treated plants than in BABA‐treated and control plants one week after treatment. After wounding, no significant treatment differences were detected, and nine weeks after wounding almost no gallocatechin was detected. For other flavonoids, no significant treatment differences were detected (Figure 6).

#### 3.1.4 Growth rate

**FIGURE 7:**
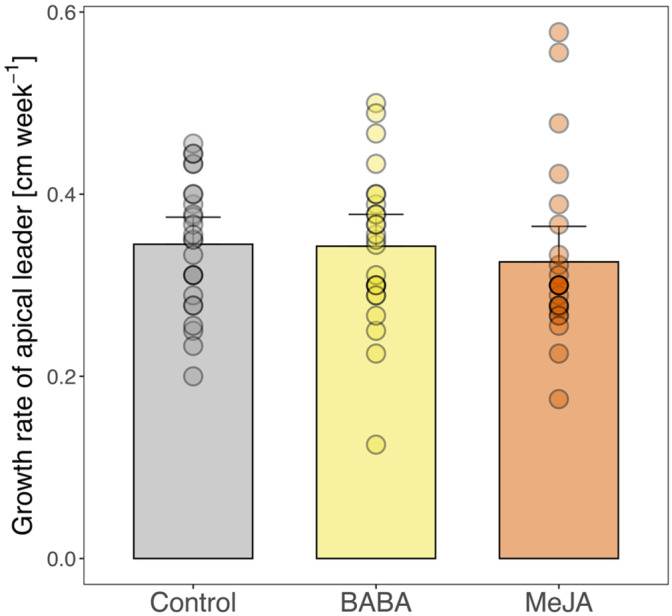
Height growth of Norway spruce plants over a 13‐week period after treatment with water and Tween (Control), β‐aminobutyric acid (BABA), or methyl jasmonate. Error bars show 95% confidence intervals and circles represent individual data points (*n* = 25). 1‐way ANOVA showed no significant differences between treatments (*p* = 0.58).

We evaluated the effect of BABA and MeJA treatment on plant growth by measuring the length of the apical leader at three different time points. Plants treated with MeJA grew 5.6% less than control plants over the 13‐week experimental period, but growth rates did not differ significantly between treatments (Figure 7).

### 3.2 Resistance to *Grosmannia penicillata*

To determine if the chemical and anatomical defense responses we quantified, contributed to plant resistance to fungal infection, we inoculated 2‐year‐old spruce plants with *G. penicillata* four weeks after the plants had been treated with BABA or MeJA. Eight weeks after inoculation, the plants were inspected for symptoms to assess plant resistance. All inoculation sites in BABA‐treated plants and 80% of the inoculation sites in control plants had discolored lesions, and these plants were thus classified as infected. In contrast, only 20% of the MeJA‐treated plants showed symptoms of infection and MeJA‐treated plants had significantly reduced infection levels compared to control and BABA‐treated plants (Table 1).

**TABLE 1:** Incidence of fungal infection eight weeks after *Grosmannia penicillata* inoculation of 2‐year‐old Norway spruce plants treated with water and Tween (Control), β‐aminobutyric acid (BABA) or methyl jasmonate (MeJA) four weeks before inoculation (n = 5 plants per treatment). Each plant was inoculated at two positions on the stem, and infection incidence is the number of plants with inoculation sites having necrotic lesions on the xylem surface. Treatments with different letters are significantly different from each other (Fisher's exact test and post‐hoc pairwise test, p < 0.05).

	Control	BABA	MeJA
**Not infected**	1	0	4
**Infected**	4	5	1
	ab	b	a

## 4 DISCUSSION

MeJA has repeatedly been demonstrated to increase resistance against pests and pathogens in Norway spruce by directly inducing or priming tree defenses (Huynh et al., 2024, Mageroy et al., 2020a). However, because MeJA application can also reduce plant growth it was of interest to test an alternative chemical priming agent (Huynh et al., 2024). BABA has been shown to be an effective priming agent in Arabidopsis (Cohen et al., 2016), but in our study BABA did not protect 2‐year‐old Norway spruce plants against infection by the bluestain fungus *G. penicillata*. Our results confirmed that MeJA induces defense responses in Norway spruce and, in contrast to previous studies, we found that MeJA increased defenses without reducing plant growth.

### 4.1 BABA did not induce resistance to *Grosmannia penicillata* infection

BABA is reported to prime defense responses in several angiosperm species against a wide range of biotic and abiotic stresses (Cohen et al., 2016). In Arabidopsis, potato, common grapevine (*Vitis vinifera*), and lettuce (*Lactuca sativa*), BABA‐induced resistance has been associated with increased callose deposition (Hamiduzzaman et al., 2005, Cohen et al., 2010, Cohen et al., 2011, Bengtsson et al., 2014, Ton and Mauch‐Mani, 2004). Callose deposition thickens cell walls and provides a tougher physical barrier to slow pathogen invasion (Voigt and Somerville, 2009). In our study, BABA did not protect Norway spruce plants against *G. penicillata* infection (Table 1). There are no studies on the role of callose in the defense of Norway spruce or other conifers. Callose has only been studied in root tips of Norway spruce plants subjected to aluminum stress (Hirano et al., 2004, Nagy et al., 2004). However, there is evidence of callose deposition in bark tissues of some conifer species (Chano et al., 2015, Krogell et al., 2012, Pearce, 1996).

Another common plant defense response to BABA treatment is accumulation of secondary metabolites, especially phenolics and phytoalexins (Piękna‐Grochala and Kępczyńska, 2013). For example, application of BABA increased concentrations of flavonoids and phenolic acids in 21‐day‐old basil (*Ocimum basilicum*) plants 15 days after treatment (Złotek et al., 2016). BABA‐treatment of Ethiopian mustard (*Brassica carinata*) was associated with an increase in total phenolics and increased resistance to the pathogen *Alternaria brassicae* (Chavan and Kamble, 2014). We did not observe increased concentrations of phenolics or other secondary metabolites in 2‐year‐old Norway spruce plants after BABA treatment or subsequent wounding. On the contrary, BABA treatment decreased concentrations of total terpenes, mostly by decreasing monoterpene concentrations in both xylem and bark tissues nine weeks after wounding and fungal inoculation. We could only find one other study quantifying effects of BABA treatment on terpenoids: Badmi and colleagues found that BABA did not induce terpenes in woodland strawberry (*Fragaria vesca*; Badmi et al., 2022). Interestingly, their study showed that BABA induced plant susceptibility to a fungal pathogen (Badmi et al., 2022). As terpenes are important defense metabolites in conifers (Gershenzon and Dudareva, 2007, Singh and Sharma, 2015, Krokene, 2015), it is not surprising that the reduced terpene concentrations we observed in BABA‐treated Norway spruce plants may lead to reduced plant resistance.

The absence of any BABA‐IR observed in Norway spruce could be due to the application methods we used. We are not aware of any published studies that have tested BABA treatment in gymnosperms. Therefore, we selected our BABA treatments based on studies performed in angiosperms and our experience with applying MeJA to Norway spruce. As the application method can affect the efficacy of BABA‐IR (Cohen et al., 2016), we tested both aboveground application (painting BABA on the bark) and soil drenching, as both methods induce resistance in angiosperms (Cohen et al., 2016). In fact, all the application methods that have been tested have been shown to induce resistance (Cohen et al., 2016). However, higher doses are required to induce resistance when BABA is applied as a foliar application (2.5–20 mM) than as a soil drench (1–5 mM; Piękna‐Grochala and Kępczyńska, 2013). Considering this, we used 50 mM BABA for bark application and 1 mM BABA for soil drenching. The relatively high dose used for bark application also takes into consideration that the outer bark of conifers is hydrophobic and quite impermeable to liquids (Franceschi et al., 2005). In the end, none of the BABA application methods we used protected Norway spruce plants against *G. penicillata* infection (Table 1) or induced any chemical or anatomical defense responses (Figure 2, 3, 4, 5 and 6).

Another important point about BABA‐IR is that efficacy can decrease greatly with increasing time between BABA application and challenge treatment (Piękna‐Grochala and Kępczyńska, 2013). This is different from MeJA application, where resistance may be induced even when plants are challenged several weeks after application (Wilkinson et al. 2022). Thus, before we can confidently conclude that BABA is ineffective in inducing Norway spruce defenses, shorter time spans between BABA application and challenge should be tested.

### 4.2 MeJA primed defense responses that can increase resistance to future attacks without reducing plant growth

Most previous studies have observed an increase in terpene concentrations in spruce bark after MeJA treatment (Erbilgin and Colgan, 2012, Erbilgin et al., 2006, Mageroy et al., 2020b, Zhao et al., 2011, Zulak et al., 2009). Interestingly, we did not observe any increase of terpenes in the bark of MeJA‐treated plants at any time point after treatment or wounding. We did, however, observe an accumulation of monoterpenes in the xylem of MeJA‐treated plants nine weeks after wounding. A similar trend was observed for traumatic resin duct (TRD) formation, indicating that TRD formation and monoterpene accumulation were primed by MeJA treatment. Our results thus resemble previous studies on mature Norway spruce trees showing primed induction of terpenes after wounding of MeJA‐treated trees (Mageroy et al., 2020b, Zhao et al., 2011). However, our observation that TRDs did not form until long after the triggering stimulus (wounding) is unique and contrary to previous studies showing that TRD formation is directly induced by MeJA treatment in Norway spruce (Martin et al., 2002). One reason for the difference in TRD formation between this study and previous studies could be genetic variance in MeJA inducibility. This variation in response to MeJA treatment among Norway spruce clones was demonstrated by Puentes and colleagues (Puentes et al., 2021). However, terpenes are known to be metabolically costly to produce, due to the high energy costs associated with chemical reduction (Gershenzon, 2017). Storage of terpenes is also metabolically costly, since it requires special storage compartments to prevent autotoxicity (Gershenzon, 1994). The primed rather than direct induction of TRDs we observed may also explain the absence of growth reduction in our experiment.

Differences in terpene accumulation between bark and sapwood have been observed previously in Norway spruce and other conifers. For example, Martin and co‐authors found that MeJA application increased terpenoid concentrations in the sapwood but not in the bark of 2‐year‐old Norway spruce plants (Martin et al., 2002). Another study in 6‐year‐old Norway spruce plants showed a similar pattern, with higher terpene levels in the sapwood following MeJA treatment but not in the bark (Schmidt et al., 2011). Two‐year‐old Scots pine (*Pinus sylvestris*) plants also showed a significant increase in monoterpene concentrations in the sapwood of plants treated with 100 mM MeJA, but no increase in the bark (Heijari et al., 2005b). In contrast, MeJA‐treatment did not alter resin concentrations in the wood of 2‐year‐old radiata pines (*Pinus radiata*) but led to significantly increased concentrations in bark tissues (Moreira et al., 2012a). Some of the variability between studies could be explained by how bark and sapwood tissues are harvested. The resin‐producing epithelial cells of developing TRDs are situated inside or near the cambial zone for the first weeks after induction (Krokene, 2015). Since this zone of fragile, thin‐walled cells forms the border between the bark and the sapwood, the cambial zone easily splits when these tissues are separated during harvesting and can be included with either bark or sapwood. To obtain more accurate results in future studies, samples should either be harvested and analyzed as whole stem sections or both sapwood and bark tissues should be included in terpene analysis.

Our analysis of phenolic compounds showed that MeJA application primed an increase in total concentrations of flavonoids and MeOH‐soluble‐tannins. The significant increase in flavonoids was mostly due to an increase in the flavan‐3‐ol catechin. Catechin concentrations were significantly higher in MeJA‐treated plants than control plants one week after MeJA treatment, returned to basal level four weeks after treatment, and then increased to even higher levels 24 h and nine weeks after wounding. MeOH‐soluble tannins, which are polymers of catechin, were also significantly higher in MeJA‐treated than in control trees after wounding. Many studies have reported an induction of catechin and its polymers upon wounding, insect attack, or fungal inoculation (Brignolas et al., 1995, Danielsson et al., 2011, Evensen et al., 2000, Jyske et al., 2020, Rohde et al., 1996). However, we are the first to show that catechin and condensed tannins may have a primed response to wounding in MeJA‐treated Norway spruce.

Flavan‐3‐ols, including the monomer catechin and its polymer condensed tannins, are effective anti‐fungal and anti‐herbivore compounds (Bueno et al., 2012, Hammerbacher et al., 2020). Even low catechin concentrations (0.1% dw) reduce the tunnelling of *Ips typographus* males by 50% (Faccoli and Schlyter, 2007). Additionally, catechin can deter bark beetle feeding and reduce beetle weight gain (Hammerbacher et al., 2019, Zhao et al., 2019). Catechin and condensed tannin can also reduce the growth of the beetle‐associated bluestain fungus *E. polonica* (Hammerbacher et al., 2014) and the root rot fungus *Heterobasidion parviporum* (Nemesio‐Gorriz et al., 2016). In poplar, increasing concentrations and re‐localization of catechin and condensed tannins contribute to increased tree resistance against infection by rust fungi in the genus *Melampsora* (Ullah et al., 2017). The same study also showed that these phenolics inhibit spore germination and hyphal growth of the rust fungus in vitro (Ullah et al., 2017). In addition, tannins have strong antioxidant properties, which can be important in plant responses to stress (Barbehenn and Peter Constabel, 2011). The documented role of catechin and its polymers in Norway spruce resistance to insects and fungi warrants more in‐depth studies of these secondary metabolites. For example, it would be interesting to study the regulation of key genes involved in flavan‐3‐ols synthesis, such as *leucoanthocyanidin reductase* (*LAR*) and *anthocyanidin reductase* *ANR*; (Tanner et al., 2003, Xie et al., 2004). In a previous transcriptomic profiling study, Mageroy and co‐workers found that *LAR* had a primed response to wounding in MeJA‐treated Norway spruce bark (Mageroy et al., 2020b).

In contrast to several previous studies showing that MeJA application reduces growth in Norway spruce (Huynh et al., 2024), we did not observe any growth differences between control and MeJA‐treated plants. MeJA‐associated growth reduction could be due to trade‐offs between plant growth and defense, such as the allocation of carbon to the formation of TRDs that are filled with metabolically costly terpenes (Wilkinson et al., 2022). In our study, the delayed induction of terpenoid defenses until after the challenge might have mitigated any growth reduction. If such a primed TRD response can be controlled experimentally it would increase the usefulness of MeJA in plant protection.

The absence of growth reduction in our study could also be due to a low application dose of MeJA (50 mM), as we painted a small volume of MeJA on the stem bark instead of spraying the whole plant, as is more commonly done. In a recently published study, application of MeJA at concentrations of 50 mM or less was shown to not reduce growth (Krokene et al., 2023). Additionally, the application of MeJA can affect the total dose that a plant receives, for example, spraying vs. painting (Huynh et al., 2024). Further research is required to find the optimal MeJA application protocol where treatment primes plant defenses without negatively affecting plant growth.

As expected, MeJA treatment reduced necrotic lesion formation in Norway spruce following *G. penicillata* inoculation (Table 1). MeJA‐induced resistance in Norway spruce against fungal infection has been observed in several studies (Kozlowski et al., 1999, Krokene et al., 2008, Zhao et al., 2011, Wilkinson et al., 2022). A recent meta‐analysis also shows that MeJA treatment significantly increases resistance to biotic attacks in different species of pine (*Pinus*) and spruce *Picea*; (Huynh et al., 2024). Thus, the results we present in this study are consistent with previous studies and strengthen the case for using MeJA application as a management tool to enhance the resistance of young spruce plants before planting. The cost and scale of treating small plants in large quantities in forest nurseries are much smaller than spraying mature trees in the forest, due to the much lower dosages and application volumes that are required (Huynh et al., 2024). However, the long‐term effects of MeJA treatment are still poorly studied, but a recent study shows that MeJA application can reduce the mortality of Norway spruce plants to pine weevil attack for up to three years after treatment (Krokene et al., 2023). Further research on the durability of MeJA‐induced resistance is needed to establish a robust protocol for using MeJA in forest nurseries.

## 5 CONCLUSIONS

In this study, we have shown that application of BABA by soil drenching did not induce resistance in Norway spruce plants to *G. penicillata* infection. In addition, the application of BABA directly on the stem did not induce anatomical or chemical defense responses or primed responses to subsequent wounding. On the contrary, BABA treatment significantly reduced terpene accumulation in the bark and xylem. In contrast, soil drenching with MeJA reduced the incidence of *G. penicillata* lesions and direct application of MeJA on the stem primed traumatic resin duct formation, and increased terpene and catechin concentrations without reducing plant growth. Based on these results, MeJA seems to have potential as a plant protection tool for young Norway spruce plants. Further and more long‐term studies are required to establish a practical protocol for tree protection using MeJA in forest nurseries.

## AUTHOR CONTRIBUTIONS

N. H. performed the lab work, analyzed the data, produced the figures, wrote the first draft, and revised the manuscript. P. K. acquired funding, contributed to the experimental design, provided guidance, and edited and revised the manuscript. V. Č. performed lab work and edited and revised the manuscript. L. N. contributed to the experimental design, provided lab facilities, provided guidance and equipment, and edited and revised the manuscript. M. M. acquired funding, contributed to the experimental design, provided guidance, and edited and revised the manuscript.

## FUNDING INFORMATION

This work was funded by the Norwegian Research Council (projects no. 249958 & 324129).

## Supporting information


**Supplementary Table S1a.** Mean concentrations (μg/g of dry weight ± SE) of terpenes in Norway spruce bark sampled 1 and 4 weeks after treatment with β‐amino butyric acid (BABA), water and Tween (Control) or methyl jasmonate (MeJA) and quantified by GC–MS analysis. For each timepoint and terpenoid, treatments with different letters (in bold) are significantly different (2‐way ANOVA followed by Tukey's HSD post hoc test, *p* < 0.05).
**Supplementary Table S1b**. Mean concentrations (μg/g^−^ of dry weight ± SE) of terpenes in Norway spruce bark sampled 24 hours and 9 weeks after wounding and quantified by GC–MS analysis. Four weeks before wounding, plants were treated with β‐amino butyric acid (BABA), water and Tween (Control) or methyl jasmonate (MeJA). For each timepoint and terpenoid, treatments with different letters (in bold) are significantly different (2‐way ANOVA followed by Tukey's HSD post hoc test, *p* < 0.05).
**Supplementary Table S2a**. Mean concentrations (μg/g of dry weight ± SE) of terpenes in Norway spruce xylem sampled 1 and 4 weeks after treatment with β‐amino butyric acid (BABA), water and Tween (Control) or methyl jasmonate (MeJA) and quantified by GC–MS analysis. There were no significant differences between treatments at these time points (2‐way ANOVA followed by Tukey's HSD post hoc test, *p* > 0.05).
**Supplementary Table S2b**. Mean concentrations (μg/g of dry weight ± SE) of terpenes in Norway spruce xylem sampled 24 hours and 9 weeks after wounding and quantified by GC–MS analysis. Four weeks before wounding, plants were treated with β‐amino butyric acid (BABA), water and Tween (Control) or methyl jasmonate (MeJA). For each timepoint and phenolic compound, treatments with different letters (in bold) are significantly different (2‐way ANOVA followed by Tukey's HSD post hoc test, *p* < 0.05).
**Supplementary Table S3a**. Mean concentrations (mg/g of dry weight ± SE) of phenolics in Norway spruce bark sampled 1 and 4 weeks after treatment with β‐amino butyric acid (BABA), water and Tween (Control) or methyl jasmonate (MeJA) and quantified by HPLC analysis. For each timepoint and phenolic compound, treatments with different letters (in bold) are significantly different (2‐way ANOVA followed by Tukey's HSD post hoc test, *p* < 0.05).
**Supplementary Table S3b**. Mean concentrations (mg/g of dry weight ± SE) of phenolics in Norway spruce bark sampled 24 hours and 9 weeks after wounding and quantified by HPLC analysis. Four weeks before wounding, plants were treated with or β‐amino butyric acid (BABA), water and Tween (Control) or methyl jasmonate (MeJA). For each timepoint and phenolic compound, treatments with different letters (in bold) are significantly different (2‐way ANOVA followed by Tukey's HSD post hoc test, *p* < 0.05).

**Supplementary Table S4.** F‐values from 2‐way ANOVAs of concentrations of terpene compounds in Norway spruce bark at different time points following treatment with different defense priming chemicals. Asterisks denote the level of significance (* *p* < 0.05, ** *p* < 0.01, *** *p* < 0.001).
**Supplementary Table S5**. F‐values from 2‐way ANOVAs of concentrations of terpene compounds in Norway spruce xylem at different time points following treatment with different defense priming chemicals. Asterisks denote the level of significance (* *p* < 0.05, ** *p* < 0.01, *** *p* < 0.001).
**Supplementary Table S6**. F‐values from 2‐way ANOVAs of concentrations of phenolic compounds in Norway spruce bark at different time points following treatment with different defense priming chemicals. Asterisks denotes the level of significance (* *p* < 0.05, ** *p* < 0.01, *** *p* < 0.001).
**Supplementary Table S7**. The High‐Performance Liquid Chromatography (HPLC) gradient used to quantify low‐molecular weight phenolics in Norway spruce bark.

**Supplementary Figure S1.** An experimental Norway spruce plant showing the apical shoot (growth measurement zone), wounding and inoculation sites, the treatment zone for application of defense priming chemicals, and the microscopy sampling zone. Note: wounding and inoculation were performed on different plants.
**Supplementary Figure S2**. Resistance phenotypes of 2‐year‐old Norway spruce plants inoculated with the bluestain fungus *Grosmannia penicillata* or mock controls 8 weeks before. Seedlings were treated with BABA or MeJA 4 weeks before inoculation or remained untreated as a control. Arrows point at the inoculation site. BABA: β‐aminobutyric acid; MeJA: methyl jasmonate.

## Data Availability

All data is provided in the supplementary. Raw data can be acquired by contacting the corresponding author.
