# Supplementary material for: β‐aminobutyric acid does not induce defenses or increase Norway spruce resistance to the bluestain fungus Grosmannia penicillata
Source: Physiol Plant. 2024 Dec 14;176(6):e70009. doi: 10.1111/ppl.70009 (PMC11645543; doi:10.1111/ppl.70009)
Supplement: Supplementary file 1 — Supplementary Table S1a. Mean concentrations (μg/g of dry weight ± SE) of terpenes in Norway spruce bark sampled 1 and 4 weeks after treatment with β‐amino butyric acid (BABA), water and Tween (Control) or methyl jasmonate (MeJA) and quantified by GC–MS analysis. For each timepoint and terpenoid, treatments with different letters (in bold) are significantly different (2‐way ANOVA followed by Tukey's HSD post hoc test, p < 0.05). Supplementary Table S1b. Mean concentrations (μg/g− of dry weight ± SE) of terpenes in Norway spruce bark sampled 24 hours and 9 weeks after wounding and quantified by GC–MS analysis. Four weeks before wounding, plants were treated with β‐amino butyric acid (BABA), water and Tween (Control) or methyl jasmonate (MeJA). For each timepoint and terpenoid, treatments with different letters (in bold) are significantly different (2‐way ANOVA followed by Tukey's HSD post hoc test, p < 0.05). Supplementary Table S2a. Mean concentrations (μg/g of dry weight ± SE) of terpenes in Norway spruce xylem sampled 1 and 4 weeks after treatment with β‐amino butyric acid (BABA), water and Tween (Control) or methyl jasmonate (MeJA) and quantified by GC–MS analysis. There were no significant differences between treatments at these time points (2‐way ANOVA followed by Tukey's HSD post hoc test, p > 0.05). Supplementary Table S2b. Mean concentrations (μg/g of dry weight ± SE) of terpenes in Norway spruce xylem sampled 24 hours and 9 weeks after wounding and quantified by GC–MS analysis. Four weeks before wounding, plants were treated with β‐amino butyric acid (BABA), water and Tween (Control) or methyl jasmonate (MeJA). For each timepoint and phenolic compound, treatments with different letters (in bold) are significantly different (2‐way ANOVA followed by Tukey's HSD post hoc test, p < 0.05). Supplementary Table S3a. Mean concentrations (mg/g of dry weight ± SE) of phenolics in Norway spruce bark sampled 1 and 4 weeks after treatment with β‐amino butyric acid (BABA [file PPL-176-e70009-s003.docx]

**Supplementary Tables**

**Supplementary Table S1a.** Mean concentrations (µg/g of dry weight ± SE) of terpenes in Norway spruce bark sampled 1 and 4 weeks after treatment with β-amino butyric acid (BABA), water and Tween (Control) or methyl jasmonate (MeJA) and quantified by GC-MS analysis. For each timepoint and terpenoid, treatments with different letters (in bold) are significantly different (2-way ANOVA followed by Tukey’s HSD post hoc test, p < 0.05).

| Type | Compound | 1 week after treatment | | |  | 4 weeks after treatment | | |
| --- | --- | --- | --- | --- | --- | --- | --- | --- |
|  |  | BABA | Control | MeJA |  | BABA | Control | MeJA |
| Monoterpenes | 3-Carene | 103.22 ± 50.11 | 20.77 ± 16.15 | 109.69 ± 55.42 |  | 262.22 ± 94.60 | 145.52 ± 95.28 | 169.45 ± 48.08 |
|  | α-Pinene | 335.85 ± 109.54 | 261.45 ± 52.23 | 295.38 ± 98.59 |  | 398.89 ± 91.39 | 238.26 ± 47.91 | 259.72 ± 72.96 |
|  | β- Pinene | 272.45 ± 91.65 | 307.43 ± 65.74 | 508.97 ± 203.65 |  | 537.91 ± 114.27 | 377.26 ± 88.29 | 315.64 ± 74.66 |
|  | β-Myrcene | 27.49 ± 10.08 | 18.30 ± 4.82 | 20.01 ± 9.59 |  | 50.45 ± 10.05 | 25.21 ± 6.22 | 13.81 ± 5.13 |
|  | β-Phellandrene | 125.33 ± 51.14 | 95.24 ± 14.99 | 128.09 ± 46.50 |  | **175.01 ± 36.89 b** | 88.86 ± 18.52 ab | **51.14 ± 16.17 a** |
|  | Camphene | **41.63 ± 14.74 b** | **9.88 ± 4.87 a** | **3.50 ± 2.27 a** |  | 28.60 ± 5.31 | 14.24 ± 3.93 | 28.30 ± 4.93 |
|  | Eucalyptol | 0.00 ± 0.00 | 0.00 ± 0.00 | 0.00 ± 0.00 |  | 0.00 ± 0.00 | 0.00 ± 0.00 | 1.75 ± 1.02 |
|  | γ-Terpinene | 0.00 ± 0.00 | 0.00 ± 0.00 | 0.00 ± 0.00 |  | 2.60 ± 1.34 | 1.47 ± 1.05 | 0.52 ± 0.52 |
|  | Limonene | 46.54 ± 20.03 | 36.40 ± 8.52 | 16.14 ± 7.43 |  | 84.47 ± 33.56 | 29.19 ± 9.15 | 35.21 ± 12.93 |
|  | *p*-Cymene | 0.00 ± 0.00 | 0.00 ± 0.00 | 0.00 ± 0.00 |  | **0.00 ± 0.00 a** | **0.00 ± 0.00 a** | **2.76 ± 0.91 b** |
|  | Sabinen | 6.00 ± 3.10 | 0.97 ± 0.97 | 3.22 ± 3.22 |  | 17.62 ± 6.20 | 7.93 ± 5.21 | 10.70 ± 3.54 |
|  | Terpinolen | 15.64 ± 6.02 | 1.99 ± 1.99 | 11.98 ± 6.48 |  | 44.50 ± 14.72 | 20.96 ± 13.03 | 10.10 ± 6.46 |
|  | ***Total monoterpenes*** | 974.15 ± 300.43 | 752.43 ± 143.18 | 1096.98 ± 368.87 |  | 1602.27 ± 246.93 | 948.9 ± 211.79 | 899.11 ± 202.38 |
| Sesqui- | Germacrene D | 137.34 ± 44.67 | 45.28 ± 6.73 | 80.20 ± 46.15 |  | 131.60 ± 65.81 | 36.94 ± 22.67 | 13.05 ± 6.10 |
|  | alfa-Gurjunene | 19.85 ± 14.87 | 7.27 ± 3.83 | 9.18 ± 4.40 |  | 14.81 ± 6.47 | 7.32 ± 3.21 | 3.83 ± 1.43 |
|  | alfa-Longipinene | 7.68 ± 7.68 | 22.52 ± 7.78 | 19.48 ± 8.48 |  | 18.78 ± 7.40 | 14.09 ± 7.95 | 22.82 ± 6.75 |
|  | ***Total sesquiterpenes*** | 164.86 ± 38.86 | 75.07 ± 10.08 | 108.85 ± 45.77 |  | 165.19 ± 65.11 | 58.35 ± 23.91 | 39.70 ± 7.27 |
| Di- | Thunbergene | 153.59 ± 29.45 | 115.77 ± 21.54 | 208.10 ± 89.14 |  | 68.24 ± 27.94 | 70.20 ± 22.19 | 36.21 ± 8.78 |
|  | Verticiol | 99.86 ± 19.22 | 77.86 ± 13.89 | 117.65 ± 71.87 |  | 38.64 ± 16.48 | 44.96 ± 15.70 | 8.04 ± 5.40 |
|  | ***Total diterpenes*** | 253.45 ± 48.58 | 193.64 ± 35.33 | 325.75 ± 158.23 |  | 106.89 ± 44.19 | 115.16 ± 36.24 | 44.25 ± 12.34 |
|  | ***Total terpenes*** | 1392.46 ± 307.91 | 1021.13 ± 181.08 | 1531.58 ± 525.43 |  | 1874.35 ± 287.54 | 1122.42 ± 219.06 | 983.06 ± 206.15 |

**Supplementary Table S1b.** Mean concentrations (µg/g^-^ of dry weight ± SE) of terpenes in Norway spruce bark sampled 24 hours and 9 weeks after wounding and quantified by GC-MS analysis. Four weeks before wounding, plants were treated with β-amino butyric acid (BABA), water and Tween (Control) or methyl jasmonate (MeJA). For each timepoint and terpenoid, treatments with different letters (in bold) are significantly different (2-way ANOVA followed by Tukey’s HSD post hoc test, p < 0.05).

| Type | Compound | 24 hours after wounding | | |  | 9 weeks after wounding | | |
| --- | --- | --- | --- | --- | --- | --- | --- | --- |
|  |  | BABA | Control | MeJA |  | BABA | Control | MeJA |
| Mono- | 3-Carene | 131.42 ± 105.19 | 121.18 ± 51.39 | 126.60 ± 61.13 |  | **84.96 ± 62.23 a** | **654.14 ± 286.80 b** | **222.45 ± 84.86 a** |
|  | α-Pinene | 352.30 ± 94.65 | 356.04 ± 70.41 | 400.79 ± 26.20 |  | 445.84 ± 91.08 | 540.33 ± 84.99 | 588.91 ± 91.98 |
|  | β- Pinene | 516.63 ± 149.79 | 651.34 ± 224.42 | 477.46 ± 122.74 |  | 651.55 ± 47.36 | 887.60 ± 180.06 | 989.07 ± 193.53 |
|  | β-Myrcene | 66.11 ± 19.06 | 54.70 ± 15.44 | 46.18 ± 5.98 |  | **76.30 ± 15.96 a** | **131.23 ± 35.71 b** | 87.94 ± 12.09 ab |
|  | β-Phellandrene | 223.28 ± 78.44 | 189.21 ± 33.26 | 141.88 ± 37.10 |  | 310.08 ± 50.21 | 437.06 ± 94.57 | 303.82 ± 37.95 |
|  | Camphene | 22.17 ± 8.07 | 25.09 ± 7.22 | 27.89 ± 2.51 |  | 22.93 ± 6.98 | 18.04 ± 3.38 | 29.29 ± 6.36 |
|  | Eucalyptol | 0.00 ± 0.00 | 0.00 ± 0.00 | 0.00 ± 0.00 |  | 0.00 ± 0.00 | 0.00 ± 0.00 | 1.30 ± 1.30 |
|  | γ-Terpinene | 1.59 ± 1.59 | 0.99 ± 0.71 | 0.75 ± 0.75 |  | **0.94 ± 0.94 a** | **12.56 ± 5.84 b** | **3.55 ± 1.46 a** |
|  | Limonene | 120.02 ± 73.99 | 58.03 ± 13.11 | 139.23 ± 44.87 |  | **59.18 ± 27.4 a** | **210.80 ± 69.50 b** | **95.17 ± 25.66 a** |
|  | *p*-Cymene | 0.00 ± 0.00 | 0.00 ± 0.00 | 0.29 ± 0.29 |  | 0.00 ± 0.00 | 1.18 ± 0.85 | 0.82 ± 0.41 |
|  | Sabinen | 11.85 ± 7.14 | 7.94 ± 4.99 | 11.14 ± 4.03 |  | **9.22 ± 3.78 a** | **62.31 ± 27.83 b** | **22.79 ± 7.81 a** |
|  | Terpinolen | 23.22 ± 13.00 | 20.22 ± 8.58 | 21.17 ± 10.96 |  | **20.58 ± 7.39 a** | **138.11 ± 57.00 b** | **41.22 ± 12.73 a** |
|  | ***Total monoterpenes*** | 1468.59 ± 394.91 | 1484.76 ± 400.08 | 1393.39 ± 127.49 |  | **1681.58 ± 221.12 a** | **3093.35 ± 797.77 b** | **2386.33 ± 316.99 ab** |
| Sesqui- | Germacrene D | 94.43 ± 47.47 | 143.98 ± 78.03 | 80.30 ± 26.79 |  | 371.42 ± 252.44 | 372.67 ± 134.67 | 341.77 ± 146.9 |
|  | α-Gurjunene | 21.04 ± 5.35 | 19.54 ± 6.81 | 22.76 ± 5.44 |  | 15.85 ± 6.35 | 26.78 ± 7.77 | 15.62 ± 6.62 |
|  | α-Longipinene | 25.36 ± 9.19 | 21.00 ± 5.96 | 27.92 ± 6.57 |  | 21.48 ± 9.43 | 44.18 ± 23.64 | 31.61 ± 9.85 |
|  | ***Total sesquiterpenes*** | 140.83 ± 36.55 | 184.52 ± 79.22 | 130.98 ± 32.01 |  | 408.76 ± 251.97 | 443.63 ± 145.71 | 389.01 ± 147.80 |
| Di- | Thunbergene | 129.19 ± 17.68 | 115.54 ± 26.43 | 108.02 ± 24.46 |  | 111.90 ± 40.71 | 101.37 ± 34.15 | 60.12 ± 22.12 |
|  | Verticiol | 64.58 ± 10.31 | 41.74 ± 19.17 | 51.07 ± 13.98 |  | 55.62 ± 20.45 | 51.31 ± 19.43 | 0.00 ± 0.00 |
|  | ***Total diterpenes*** | 193.78 ± 27.64 | 157.28 ± 36.96 | 159.09 ± 37.91 |  | 167.52 ± 60.70 | 152.67 ± 53.34 | 60.12 ± 22.12 |
|  | ***Total terpenes*** | 1803.19 ± 348.49 | 1826.56 ± 406.86 | 1683.46 ± 117.06 |  | **2257.85 ± 408.10 a** | **3689.65 ± 856.94 b** | **2835.45 ± 420.80 ab** |

**Supplementary Table S2a.** Mean concentrations (µg/g of dry weight ± SE) of terpenes in Norway spruce xylem sampled 1 and 4 weeks after treatment with β-amino butyric acid (BABA), water and Tween (Control) or methyl jasmonate (MeJA) and quantified by GC-MS analysis. There were no significant differences between treatments at these time points (2-way ANOVA followed by Tukey’s HSD post hoc test, p > 0.05).

| Type | Compound | 1 week after treatment | | |  | 4 weeks after treatment | | |
| --- | --- | --- | --- | --- | --- | --- | --- | --- |
|  |  | BABA | Control | MeJA |  | BABA | Control | MeJA |
| Monoterpenes | 3-Carene | 11.02 ± 6.71 | 0.00 ± 0.00 | 59.73 ± 58.55 |  | 48.28 ± 42.66 | 0.09 ± 0.09 | 10.92 ± 7.41 |
|  | α-Pinene | 94.81 ± 61.55 | 0.27 ± 0.27 | 38.49 ± 17.49 |  | 34.07 ± 18.74 | 63.36 ± 35.84 | 52.66 ± 35.22 |
|  | β- Pinene | 99.43 ± 66.35 | 1.07 ± 0.80 | 64.53 ± 28.16 |  | 64.28 ± 36.28 | 125.87 ± 75.75 | 47.81 ± 26.99 |
|  | β-Myrcene | 9.30 ± 7.41 | 0.00 ± 0.00 | 7.66 ± 4.68 |  | 3.48 ± 3.01 | 12.62 ± 7.38 | 2.58 ± 1.69 |
|  | β-Phellandrene | 37.15 ± 29.05 | 0.18 ± 0.18 | 27.00 ± 14.54 |  | 16.17 ± 10.66 | 60.79 ± 37.76 | 7.71 ± 4.79 |
|  | Camphene | 7.74 ± 5.89 | 0.00 ± 0.00 | 1.86 ± 1.42 |  | 3.52 ± 2.51 | 2.52 ± 1.38 | 5.24 ± 3.09 |
|  | Eucalyptol | 0.00 ± 0.00 | 0.00 ± 0.00 | 1.67 ± 0.68 |  | 0.00 ± 0.00 | 0.00 ± 0.00 | 0.42 ± 0.42 |
|  | γ-Terpinene | 0.13 ± 0.13 | 0.00 ± 0.00 | 0.85 ± 0.85 |  | 0.75 ± 0.75 | 0.00 ± 0.00 | 0.00 ± 0.00 |
|  | Limonene | 8.88 ± 8.00 | 0.00 ± 0.00 | 8.60 ± 5.63 |  | 3.50 ± 1.9 | 12.5 ± 7.48 | 5.35 ± 3.63 |
|  | *p*-Cymene | 0.00 ± 0.00 | 0.00 ± 0.00 | 0.00 ± 0.00 |  | 0.00 ± 0.00 | 0.00 ± 0.00 | 0.11 ± 0.11 |
|  | Sabinen | 2.17 ± 2.17 | 0.00 ± 0.00 | 5.70 ± 5.10 |  | 3.35 ± 3.35 | 2.31 ± 2.31 | 1.42 ± 0.92 |
|  | Terpinolen | 2.88 ± 2.14 | 0.00 ± 0.00 | 8.22 ± 7.94 |  | 7.68 ± 7.21 | 0.78 ± 0.63 | 1.09 ± 0.75 |
|  | ***Total monoterpenes*** | 273.51 ± 187.67 | 1.52 ± 1.24 | 224.32 ± 133.72 |  | 185.08 ± 120.39 | 280.83 ± 166.72 | 135.31 ± 77.83 |
| Sesqui- | Germacrene D | 7.19 ± 4.12 | 0.00 ± 0.00 | 3.64 ± 2.87 |  | 23.11 ± 21.91 | 0.65 ± 0.65 | 0.53 ± 0.42 |
|  | alfa-Gurjunene | 5.09 ± 4.56 | 0.00 ± 0.00 | 0.00 ± 0.00 |  | 0.00 ± 0.00 | 0.00 ± 0.00 | 0.00 ± 0.00 |
|  | alfa-Longipinene | 0.61 ± 0.61 | 0.00 ± 0.00 | 1.55 ± 1.54 |  | 0.99 ± 0.7 | 1.73 ± 1.28 | 0.57 ± 0.35 |
|  | ***Total sesquiterpenes*** | 12.90 ± 9.20 | 0.00 ± 0.00 | 5.19 ± 3.22 |  | 24.1 ± 22.56 | 2.39 ± 1.22 | 1.1 ± 0.73 |
| Di- | Thunbergene | 19.34 ± 8.52 | 0.12 ± 0.12 | 7.71 ± 4.9 |  | 2.23 ± 1.45 | 21.22 ± 13.33 | 0.75 ± 0.75 |
|  | Verticiol | 18.67 ± 8.12 | 0.00 ± 0.00 | 8.25 ± 4.52 |  | 1.46 ± 1.46 | 21.32 ± 13.09 | 0.83 ± 0.83 |
|  | ***Total diterpenes*** | 38.02 ± 16.63 | 0.12 ± 0.12 | 15.96 ± 9.39 |  | 3.68 ± 2.8 | 42.54 ± 26.41 | 1.57 ± 1.57 |
|  | ***Total terpenes*** | 324.42 ± 212.6 | 1.65 ± 1.36 | 245.47 ± 137.73 |  | 212.86 ± 142.82 | 325.76 ± 193.54 | 137.98 ± 79.29 |

**Supplementary Table S2b.** Mean concentrations (µg/g of dry weight ± SE) of terpenes in Norway spruce xylem sampled 24 hours and 9 weeks after wounding and quantified by GC-MS analysis. Four weeks before wounding, plants were treated with β-amino butyric acid (BABA), water and Tween (Control) or methyl jasmonate (MeJA). For each timepoint and phenolic compound, treatments with different letters (in bold) are significantly different (2-way ANOVA followed by Tukey’s HSD post hoc test, p < 0.05).

| Type | Compound | 24 hours after wounding | | |  | 9 weeks after wounding | | |
| --- | --- | --- | --- | --- | --- | --- | --- | --- |
|  |  | BABA | Control | MeJA |  | BABA | Control | MeJA |
| Monoterpenes | 3-Carene | 0.46 ± 0.32 | 14.33 ± 11.2 | 4.24 ± 1.53 |  | **2.19 ± 1.43 a** | **199.36 ± 122.54 b** | 98.47 ± 79.09 ab |
|  | α-Pinene | 18.81 ± 8.8 | 26.65 ± 15.39 | 184.08 ± 99.48 |  | **112.79 ± 31.87 a** | **490.91 ± 170.89 b** | **1249.58 ± 165.98 c** |
|  | β- Pinene | 27.73 ± 12.73 | 58.73 ± 32.62 | 143.76 ± 74.29 |  | **157.57 ± 51.93 a** | **584.2 ± 244.39 b** | **1098.63 ± 278.42 c** |
|  | β-Myrcene | 2.77 ± 1.32 | 6.56 ± 4.92 | 12.78 ± 6.61 |  | **16.43 ± 6.52 a** | **80.52 ± 30.55 b** | **138.02 ± 29.24 c** |
|  | β-Phellandrene | 10.92 ± 5.68 | 22.30 ± 16.32 | 35.95 ± 19.22 |  | **59.94 ± 25.57 a** | **215.39 ± 85.28 b** | **320.16 ± 83.78 b** |
|  | Camphene | 1.14 ± 0.57 | 1.49 ± 1.19 | 9.48 ± 5.24 |  | **4.64 ± 1.57 a** | **15.91 ± 6.08 a** | **43.17 ± 3.91 b** |
|  | Eucalyptol | 0.00 ± 0.00 | 0.00 ± 0.00 | 0.49 ± 0.49 |  | 0.00 ± 0.00 | 0.00 ± 0.00 | 2.51 ± 2.51 |
|  | γ-Terpinene | 0.00 ± 0.00 | 0.18 ± 0.18 | 0.00 ± 0.00 |  | **0.00 ± 0.00 a** | **4.40 ± 2.58 b** | 2.31 ± 1.38 ab |
|  | Limonene | 3.46 ± 2.13 | 5.67 ± 4.62 | 17.53 ± 7.78 |  | **8.43 ± 2.4 a** | **126.54 ± 47.98 b** | **105.13 ± 19.27 b** |
|  | *p*-Cymene | 0.00 ± 0.00 | 0.00 ± 0.00 | 0.00 ± 0.00 |  | **0.00 ± 0.00 a** | **0.44 ± 0.31 b** | 0.27 ± 0.18 ab |
|  | Sabinen | 0.00 ± 0.00 | 2.17 ± 1.57 | 2.38 ± 2.09 |  | **1.01 ± 0.86 a** | **31.07 ± 20.06 b** | **51.86 ± 12.46 b** |
|  | Terpinolen | 0.33 ± 0.18 | 2.58 ± 2.2 | 1.89 ± 1.15 |  | **2.54 ± 1.12 a** | **40.88 ± 20.9 b** | **40.25 ± 13.92 b** |
|  | ***Total monoterpenes*** | 65.63 ± 30.11 | 140.65 ± 88.05 | 412.58 ± 203.07 |  | **365.55 ± 116.85 a** | **1789.62 ± 678.84 b** | **3150.36 ± 560.85 c** |
| Sesqui- | Germacrene D | 3.23 ± 1.71 | 8.07 ± 6.65 | 2.14 ± 1.44 |  | 98.78 ± 91.48 | 170.17 ± 94.90 | 76.78 ± 22.88 |
|  | alfa-Gurjunene | 0.03 ± 0.03 | 0.99 ± 0.99 | 1.01 ± 1.01 |  | **2.15 ± 1.41 a** | 10.03 ± 6.50 ab | **12.44 ± 2.81 b** |
|  | alfa-Longipinene | 0.30 ± 0.30 | 1.03 ± 0.93 | 1.11 ± 0.4 |  | **1.47 ± 0.54 a** | **13.08 ± 6.29 b** | **18.69 ± 8.15 b** |
|  | ***Total sesquiterpenes*** | 3.55 ± 1.71 | 10.09 ± 8.56 | 4.27 ± 1.88 |  | 102.39 ± 92.3 | 193.55 ± 100.48 | 107.91 ± 24.92 |
| Di- | Thunbergene | 5.65 ± 2.64 | 9.55 ± 5.65 | 14.24 ± 7.71 |  | 38.30 ± 20.83 ab | **109.92 ± 81.54 b** | **21.60 ± 4.79 a** |
|  | Verticiol | 5.51 ± 2.51 | 9.22 ± 5.42 | 12.44 ± 7.93 |  | 31.63 ± 19.72 ab | **97.84 ± 72.95 b** | **14.00 ± 4.97 a** |
|  | ***Total diterpenes*** | 11.16 ± 5.15 | 18.77 ± 11.06 | 26.67 ± 15.62 |  | 69.93 ± 40.42 ab | **207.76 ± 154.49 b** | **35.61 ± 9.02 a** |
|  | ***Total terpenes*** | 80.34 ± 35.19 | 169.52 ± 107.00 | 443.52 ± 220.22 |  | **537.87 ± 234.81 a** | **2190.92 ± 832.32 b** | **3293.88 ± 582.77 b** |

**Supplementary Table S3a.** Mean concentrations (mg/g of dry weight ± SE) of phenolics in Norway spruce bark sampled 1 and 4 weeks after treatment with β-amino butyric acid (BABA), water and Tween (Control) or methyl jasmonate (MeJA) and quantified by HPLC analysis. For each timepoint and phenolic compound, treatments with different letters (in bold) are significantly different (2-way ANOVA followed by Tukey’s HSD post hoc test, p < 0.05).

|  |  | 1 week after treatment | | |  | 4 weeks after treatment | | |
| --- | --- | --- | --- | --- | --- | --- | --- | --- |
| Type | Compound | BABA | Control | MeJA |  | BABA | Control | MeJA |
| ***Acetophenon*** | Picein | 0.16 ± 0.06 | 0.32 ± 0.19 | 0.24 ± 0.07 |  | **0.18 ± 0.04 a** | **0.58 ± 0.20 b** | **0.12 ± 0.06 a** |
| Flavonoids | Gallocatechin | **4.06 ± 0.22 a** | **3.05 ± 0.54 a** | **1.33 ± 0.58 b** |  | **1.59 ± 0.24 b** | 1.13 ± 0.50 ab | **0.11 ± 0.08 a** |
|  | Catechin | 1.84 ± 0.34 ab | **1.56 ± 0.30 a** | **3.80 ± 0.91 b** |  | 1.28 ± 0.22 | 1.41 ± 0.20 | 1.44 ± 0.57 |
|  | Dihydromyricetin 1 | 0.02 ± 0.01 | 0.01 ± 0.01 | 0.02 ± 0.01 |  | 0.00 ± 0.00 | 0.01 ± 0.01 | 0.00 ± 0.00 |
|  | Dihydromyricetin 2 | 0.12 ± 0.02 | 0.18 ± 0.06 | 0.20 ± 0.02 |  | 0.15 ± 0.02 | 0.19 ± 0.03 | 0.18 ± 0.06 |
|  | Dihydromyricetin 3 | 0.02 ± 0.01 | 0.03 ± 0.02 | 0.04 ± 0.03 |  | 0.02 ± 0.01 | 0.02 ± 0.01 | 0.04 ± 0.02 |
|  | Dihydromyricetin 4 | 0.04 ± 0.02 | 0.10 ± 0.02 | 0.08 ± 0.02 |  | 0.06 ± 0.01 | 0.06 ± 0.01 | 0.04 ± 0.01 |
|  | Quercetin glycoside | 0.65 ± 0.15 | 0.40 ± 0.12 | 0.72 ± 0.30 |  | 0.55 ± 0.12 | 0.76 ± 0.09 | 0.65 ± 0.15 |
|  | Monocoumaryl astragalin 1 | 0.17 ± 0.01 | 0.29 ± 0.14 | 0.18 ± 0.06 |  | 0.25 ± 0.03 | 0.34 ± 0.06 | 0.34 ± 0.05 |
|  | Monocoumaryl astragalin 2 | 0.00 ± 0.00 | 0.01 ± 0.01 | 0.02 ± 0.01 |  | **0.01 ± 0.01 a** | 0.02 ± 0.01 ab | **0.05 ± 0.02 b** |
|  | Dicoumaryl astragallin | 2.07 ± 0.29 | 3.47 ± 1.22 | 3.43 ± 0.48 |  | 4.22 ± 0.62 | 4.39 ± 0.42 | 5.55 ± 0.71 |
| ***Total flavonoids*** | | 8.99 ± 0.65 | 9.11 ± 1.39 | 9.84 ± 1.13 |  | 8.14 ± 0.48 | 8.33 ± 0.49 | 8.41 ± 0.97 |
| Stilbenes | Piceatannol glycoside | 4.12 ± 0.90 | 4.66 ± 0.79 | 3.16 ± 0.25 |  | 2.91 ± 0.59 ab | **4.69 ± 0.69 b** | **1.18 ± 0.45 a** |
|  | Piceatannol aglycon | **0.12 ± 0.04 b** | 0.08 ± 0.03 ab | **0.00 ± 0.00 a** |  | 0.06 ± 0.03 | 0.08 ± 0.04 | 0.04 ± 0.02 |
|  | Resveratrol glycoside | 0.00 ± 0.00 | 0.00 ± 0.00 | 0.00 ± 0.00 |  | 0.0008 ± 0.00 | 0.0014 ± 0.00 | 0.0003 ± 0.00 |
|  | Iso-rhapontin glycoside | 13.71 ± 3.82 | 13.01 ± 3.45 | 14.79 ± 1.36 |  | 11.61 ± 2.73 ab | **14.85 ± 3.77 b** | **4.07 ± 1.77 a** |
|  | E-astringin | 0.23 ± 0.07 | 0.30 ± 0.10 | 0.44 ± 0.09 |  | 0.19 ± 0.06 ab | **0.32 ± 0.12 b** | **0.09 ± 0.02 a** |
|  | Piceatannol | 0.03 ± 0.02 | 0.02 ± 0.00 | 0.01 ± 0.01 |  | 0.03 ± 0.01 | 0.02 ± 0.01 | 0.01 ± 0.01 |
|  | Unknown stilbene 1 | 0.03 ± 0.01 | 0.05 ± 0.01 | 0.03 ± 0.01 |  | 0.04 ± 0.01 | 0.05 ± 0.01 | 0.06 ± 0.01 |
|  | Unknown stilbene 2 | 0.00 ± 0.00 | 0.01 ± 0.00 | 0.01 ± 0.00 |  | 0.01 ± 0.00 | 0.02 ± 0.00 | 0.02 ± 0.00 |
| ***Total stilbenes*** | | 18.23 ± 4.70 | 18.13 ± 3.80 | 18.44 ± 1.52 |  | 14.85 ± 3.27 ab | **20.04 ± 4.11 b** | **5.47 ± 2.22 a** |
| ***Total low-molecular-weight phenolic compounds*** | | 27.39 ± 5.37 | 27.56 ± 5.10 | 28.51 ± 2.25 |  | 23.17 ± 3.43 ab | **28.95 ± 4.25 b** | **14.00 ± 2.94 a** |
| MeOH-insoluble condensed tannins | | **37.54 ± 4.49 b** | 24.14 ± 3.45 ab | **22.89 ± 2.57 a** |  | **23.64 ± 2.76 a** | **42.00 ± 5.21 b** | **21.93 ± 2.62 a** |
| MeOH-soluble condensed tannins | | 22.14 ± 4.59 | 30.15 ± 2.71 | 34.37 ± 6.52 |  | 19.60 ± 4.49 ab | **27.00 ± 3.34 b** | **12.15 ± 4.29 a** |

**Supplementary Table S3b.** Mean concentrations (mg/g of dry weight ± SE) of phenolics in Norway spruce bark sampled 24 hours and 9 weeks after wounding and quantified by HPLC analysis. Four weeks before wounding, plants were treated with or β-amino butyric acid (BABA), water and Tween (Control) or methyl jasmonate (MeJA). For each timepoint and phenolic compound, treatments with different letters (in bold) are significantly different (2-way ANOVA followed by Tukey’s HSD post hoc test, p < 0.05).

|  |  | 24 hours after wounding | | |  | 9 weeks after wounding | | |
| --- | --- | --- | --- | --- | --- | --- | --- | --- |
| Type | Compound | BABA | Control | MeJA | BABA | | Control | MeJA |
| *Acetophenon* | Picein | 0.14 ± 0.04 | 0.23 ± 0.06 | 0.17 ± 0.03 | 0.18 ± 0.05 | | 0.11 ± 0.04 | 0.11 ± 0.02 |
| Flavonoids | Gallocatechin | 3.02 ± 0.82 | 1.80 ± 0.27 | 2.09 ± 0.48 | 0.00 ± 0.00 | | 0.00 ± 0.00 | 0.30 ± 0.30 |
|  | Catechin | **2.36 ± 0.18 a** | **1.96 ± 0.31 a** | **5.84 ± 0.80 b** | **2.42 ± 0.27 a** | | **3.55 ± 0.49 a** | **7.13 ± 0.94 b** |
|  | Dihydromyricetin 1 | 0.00 ± 0.00 | 0.00 ± 0.00 | 0.00 ± 0.00 | **0.01 ± 0.01 a** | | **0.02 ± 0.02 a** | **0.07 ± 0.03 b** |
|  | Dihydromyricetin 2 | 0.13 ± 0.03 | 0.18 ± 0.02 | 0.22 ± 0.03 | 0.17 ± 0.01 | | 0.14 ± 0.02 | 0.15 ± 0.01 |
|  | Dihydromyricetin 3 | 0.05 ± 0.01 | 0.03 ± 0.02 | 0.07 ± 0.02 | 0.01 ± 0.01 | | 0.05 ± 0.01 | 0.06 ± 0.01 |
|  | Dihydromyricetin 4 | 0.08 ± 0.03 | 0.06 ± 0.03 | 0.07 ± 0.03 | 0.10 ± 0.02 | | 0.10 ± 0.03 | 0.08 ± 0.03 |
|  | Quercetin glycoside | 0.33 ± 0.09 | 0.68 ± 0.11 | 0.62 ± 0.15 | 0.65 ± 0.15 | | 0.36 ± 0.08 | 0.39 ± 0.08 |
|  | Monocoumaryl astragalin 1 | 0.24 ± 0.07 | 0.21 ± 0.06 | 0.29 ± 0.03 | 0.19 ± 0.08 | | 0.08 ± 0.02 | 0.08 ± 0.03 |
|  | Monocoumaryl astragalin 2 | 0.01 ± 0.01 | 0.02 ± 0.01 | 0.02 ± 0.01 | 0.01 ± 0.01 | | 0.02 ± 0.02 | 0.01 ± 0.01 |
|  | Dicoumaryl astragallin | 2.73 ± 0.56 | 3.73 ± 1.14 | 4.10 ± 0.61 | 2.63 ± 0.97 | | 1.09 ± 0.28 | 1.29 ± 0.23 |
| *Total flavonoids* | | **8.95 ± 0.5 a** | **8.67 ± 1.24 a** | **13.33 ± 1.28 b** | **6.19 ± 1.24 a** | | **5.41 ± 0.74 a** | **9.57 ± 0.96 b** |
| Stilbenes | Piceatannol glycoside | 3.83 ± 0.75 | 2.97 ± 0.26 | 4.09 ± 1.27 | **5.32 ± 0.62 a** | | **10.45 ± 1.7511 b** | **8.55 ± 1.36 a** |
|  | Piceatannol aglycon | 0.00 ± 0.00 | 0.03 ± 0.03 | 0.07 ± 0.04 | 0.08 ± 0.03 | | 0.15 ± 0.02 | 0.14 ± 0.03 |
|  | Resveratrol glycoside | 0.00 ± 0.00 | 0.00 ± 0.00 | 0.00 ± 0.00 | **0.0014 ± 0.00 a** | | **0.0035 ± 0.00 b** | **0.003 ± 0.00 b** |
|  | Iso-rhapontin glycoside | 11.67 ± 1.34 | 10.83 ± 1.86 | 11.43 ± 2.64 | 18.44 ± 1.51 | | 27.85 ± 3.74 | 24.09 ± 3.62 |
|  | E-astringin | **0.57 ± 0.09 b** | 0.32 ± 0.04 ab | **0.25 ± 0.05 a** | 0.20 ± 0.05 | | 0.29 ± 0.08 | 0.31 ± 0.10 |
|  | Piceatannol | 0.03 ± 0.00 | 0.02 ± 0.01 | 0.02 ± 0.01 | 0.08 ± 0.02 ab | | **0.12 ± 0.02 b** | **0.075 ± 0.01 a** |
|  | Unknown stilbene 1 | 0.04 ± 0.01 | 0.05 ± 0.01 | 0.04 ± 0.01 | 0.03 ± 0.01 | | 0.02 ± 0.01 | 0.02 ± 0.00 |
|  | Unknown stilbene 2 | **0.00 ± 0.00 a** | 0.01 ± 0.01 ab | **0.02 ± 0.00 b** | 0.00 ± 0.00 | | 0.00 ± 0.00 | 0.00 ± 0.00 |
| *Total stilbenes* | | 16.14 ± 2.11 | 14.22 ± 2.06 | 15.92 ± 3.80 | **24.15 ± 1.57 a** | | **38.89 ± 5.29 b** | 33.20 ± 4.53 ab |
| *Total low-molecular-weight phenolic compounds* | | 25.22 ± 2.60 | 23.12 ± 3.16 | 29.43 ± 4.14 | 30.53 ± 1.20 | | 44.40 ± 5.52 | 42.87 ± 4.01 |
| MeOH-insoluble tannins | | 38.02 ± 4.77 | 29.61 ± 3.18 | 24.80 ± 1.99 | 44.62 ± 4.76 | | 30.46 ± 5.41 | 33.55 ± 1.83 |
| MeOH-soluble tannins | | 35.58 ± 1.06 | 29.25 ± 1.68 | 38.98 ± 1.89 b | **45.49 ± 5.93 a** | | 56.95 ± 1.24 ab | **65.23 ± 3.35 b** |
