# Supplementary material for: β‐aminobutyric acid does not induce defenses or increase Norway spruce resistance to the bluestain fungus Grosmannia penicillata
Source: Physiol Plant. 2024 Dec 14;176(6):e70009. doi: 10.1111/ppl.70009 (PMC11645543; doi:10.1111/ppl.70009)
Supplement: Supplementary file 2 — Supplementary Table S4. F‐values from 2‐way ANOVAs of concentrations of terpene compounds in Norway spruce bark at different time points following treatment with different defense priming chemicals. Asterisks denote the level of significance (* p < 0.05, ** p < 0.01, *** p < 0.001). Supplementary Table S5. F‐values from 2‐way ANOVAs of concentrations of terpene compounds in Norway spruce xylem at different time points following treatment with different defense priming chemicals. Asterisks denote the level of significance (* p < 0.05, ** p < 0.01, *** p < 0.001). Supplementary Table S6. F‐values from 2‐way ANOVAs of concentrations of phenolic compounds in Norway spruce bark at different time points following treatment with different defense priming chemicals. Asterisks denotes the level of significance (* p < 0.05, ** p < 0.01, *** p < 0.001). Supplementary Table S7. The High‐Performance Liquid Chromatography (HPLC) gradient used to quantify low‐molecular weight phenolics in Norway spruce bark. [file PPL-176-e70009-s001.docx]

**Supplementary Table S4.** F-values from 2-way ANOVAs of concentrations of terpene compounds in Norway spruce bark at different time points following treatment with different defense priming chemicals. Asterisks denote the level of significance (* p < 0.05, ** p < 0.01, *** p < 0.001).

| Compound | Treatment  (*F_2,60_*) | Time point (*F_3,60_*) | Treatment × Time point (*F_6,60_*) |
| --- | --- | --- | --- |
| 3-Carene | 0.56 | 2.75 | 2.51 * |
| α-Pinene | 0.38 | 5.14 ** | 0.70 |
| β-Pinene | 0.09 | 7.89 *** | 1.22 |
| β-Myrcene | 2.04 | 19.54 *** | 1.86 |
| β-Phellandrene | 2.41 | 20.27 *** | 1.43 |
| Camphene | 3.66 * | 0.59 | 2.74 * |
| Eucalyptol | 3.09 | 0.80 | 0.73 |
| γ-Terpinene | 2.30 | 5.60 ** | 3.11 * |
| Limonene | 0.19 | 4.82 ** | 2.55 * |
| *p*-Cymene | 6.91 ** | 2.69 | 2.98 * |
| Sabinen | 0.79 | 5.37 ** | 3.05 * |
| Terpinolen | 1.91 | 5.37 ** | 3.72 ** |
| ***Total monoterpenes*** | 0.17 | 10.87 *** | 2.02 |
| α-Gurjunene | 0.97 | 3.01 * | 0.74 |
| α-Longipinene | 0.55 | 1.48 | 0.52 |
| Germacrene D | 0.55 | 7.22 *** | 0.18 |
| ***Total sesquiterpenes*** | 0.52 | 8.20 *** | 0.23 |
| Thunbergene | 0.31 | 5.79 ** | 0.93 |
| Verticiol | 1.19 | 5.68 ** | 0.93 |
| ***Total diterpenes*** | 0.62 | 5.95 ** | 0.95 |
| ***Total terpenes*** | 0.35 | 12.41 *** | 1.91 |

**Supplementary Table S5.** F-values from 2-way ANOVAs of concentrations of terpene compounds in Norway spruce xylem at different time points following treatment with different defense priming chemicals. Asterisks denote the level of significance (* p < 0.05, ** p < 0.01, *** p < 0.001).

| Compound | Treatment  (*F_2,48_*) | Time point (*F_3,48_*) | Treatment × Time point (*F_6,48_*) |
| --- | --- | --- | --- |
| 3-Carene | 0.69 | 2.42 | 1.48 |
| α-Pinene | 17.29 *** | 37.85 *** | 12.72 *** |
| β-Pinene | 4.79 * | 16.65 *** | 4.16 ** |
| β-Myrcene | 6.12 ** | 22.78 *** | 5.36 *** |
| β-Phellandrene | 3.02 | 15.01 *** | 3.05 * |
| Camphene | 12.34 *** | 20.10 *** | 8.39 *** |
| Eucalyptol | 3.61 * | 0.56 | 0.56 |
| γ-Terpinene | 1.06 | 3.77 * | 1.77 |
| Limonene | 4.63 * | 16.26 *** | 3.99 ** |
| *p*-Cymene | 1.18 | 3.20 * | 1.07 |
| Sabinen | 3.68 | 9.79 *** | 3.11 * |
| Terpinolen | 1.61 | 7.49 *** | 2.18 ** |
| ***Total monoterpenes*** | 7.38 ** | 23.83 *** | 6.04 *** |
| α-Gurjunene | 0.39 | 6.99 *** | 1.92 |
| α-Longipinene | 2.39 | 8.34 *** | 1.99 |
| Germacrene D | 0.37 | 5.89 ** | 0.43 |
| ***Total sesquiterpenes*** | 0.31 | 7.35 *** | 0.47 |
| Thunbergene | 1.03 | 2.73 | 0.96 |
| Verticiol | 1.16 | 2.27 | 1.05 |
| ***Total diterpenes*** | 1.09 | 2.51 | 1.00 |
| ***Total terpenes*** | 5.17 ** | 22.63 *** | 4.53 ** |

**Supplementary Table S6.** F-values from 2-way ANOVAs of concentrations of phenolic compounds in Norway spruce bark at different time points following treatment with different defense priming chemicals. Asterisks denotes the level of significance (* p < 0.05, ** p < 0.01, *** p < 0.001).

| Compound | Treatment  (*F_2,60_*) | Time point  (*F_3,60_*) | Treatment × Time point  (*F_6,60_*) |
| --- | --- | --- | --- |
| Picein | 4.2406 * | 1.3181 | 1.5061 |
|  |  |  |  |
| Gallocatechin | 10.391 *** | 27.354 *** | 2.886 * |
| (+) Catechin | 21.996 *** | 21.388 *** | 5.066 *** |
| Dihydromyricetin 1 | 2.619 | 6.583 *** | 2.389 * |
| Dihydromyricetin 2 | 1.262 | 0.242 | 0.532 |
| Dihydromyricetin 3 | 3.328 * | 1.392 | 0.644 |
| Dihydromyricetin 4 | 0.393 | 1.991 | 0.832 |
| Quercetin glycoside | 0.135 | 1.006 | 1.404 |
| Monocoumaryl astragallin 1 | 0.253 | 6.032 ** | 1.105 |
| Monocoumaryl astragallin 2 | 3.020 | 1.525 | 0.644 |
| Dicoumaryl astragallin | 1.296 | 11.622 *** | 1.189 |
| *Total flavonoids* | 6.111 *** | 5.624 ** | 1.873 |
| Piceatannol glycoside | 5.274 ** | 23.023 *** | 3.251 ** |
| Resveratrol glycoside | 2.077 | 13.462 *** | 2.094 |
| Iso-rhapontin glycoside | 2.268 | 13.469 *** | 1.868 |
| E-astringin | 0.665 | 3.442 * | 2.828 * |
| Piceatannol aglycon | 0.793 | 4.250 ** | 1.857 |
| Piceatannol | 1.248 | 27.606 *** | 1.211 |
| Unknown stilbene 1 | 1.170 | 3.890 * | 1.03 |
| Unknown stilbene 2 | 2.461 | 9.712 *** | 0.794 |
| *Total stilbenes* | 3.284 * | 17.779 *** | 2.396 * |
| *Total low-molecular-weight phenolic compounds* | 1.703 | 12.098 *** | 2.600 * |
| MeOH-soluble tannins | 2.011 | 46.917 *** | 3.755 * |
| MeOH-insoluble tannins | 6.804 ** | 2.615 | 4.922 *** |

**Supplementary Table S7.** The High-Performance Liquid Chromatography (HPLC) gradient used to quantify low-molecular weight phenolics in Norway spruce bark.

| Time (min) | Solution A (%) | Solution B (%) |
| --- | --- | --- |
| Initial | 100 | 0 |
| 5 | 100 | 0 |
| 10 | 85 | 15 |
| 20 | 70 | 30 |
| 40 | 50 | 50 |
| 45 | 50 | 50 |
| Rinsing | 0 | 100 |
| Equilibration | 100 | 0 |
