# Supplementary material for: β‐aminobutyric acid does not induce defenses or increase Norway spruce resistance to the bluestain fungus Grosmannia penicillata
Source: Physiol Plant. 2024 Dec 14;176(6):e70009. doi: 10.1111/ppl.70009 (PMC11645543; doi:10.1111/ppl.70009)
Supplement: Supplementary file 3 — Supplementary Figure S1. An experimental Norway spruce plant showing the apical shoot (growth measurement zone), wounding and inoculation sites, the treatment zone for application of defense priming chemicals, and the microscopy sampling zone. Note: wounding and inoculation were performed on different plants. Supplementary Figure S2. Resistance phenotypes of 2‐year‐old Norway spruce plants inoculated with the bluestain fungus Grosmannia penicillata or mock controls 8 weeks before. Seedlings were treated with BABA or MeJA 4 weeks before inoculation or remained untreated as a control. Arrows point at the inoculation site. BABA: β‐aminobutyric acid; MeJA: methyl jasmonate. [file PPL-176-e70009-s002.docx]

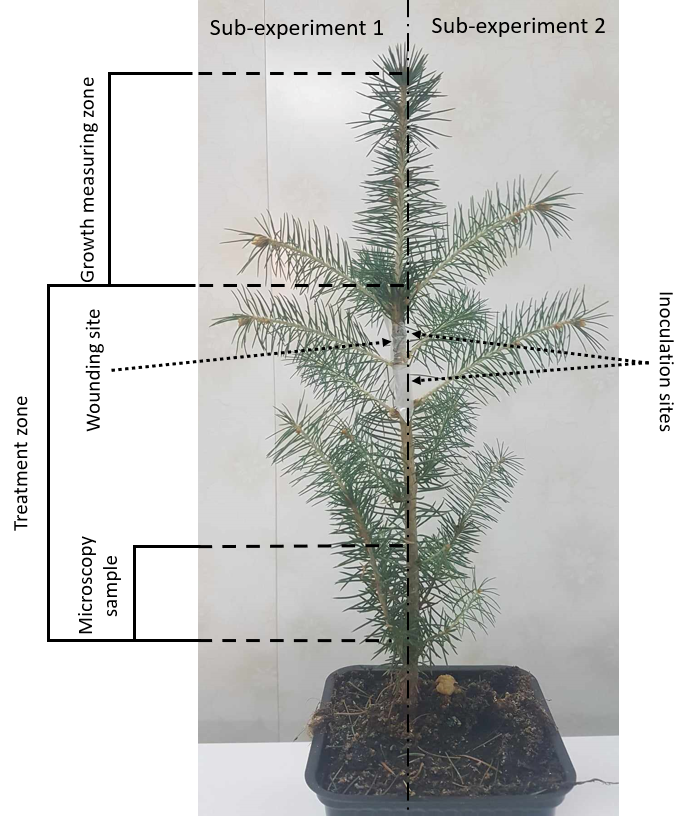
Supplementary Figure S1. An experimental Norway spruce plant showing the apical shoot (growth measurement zone), wounding and inoculation sites, the treatment zone for application of defense priming chemicals, and the microscopy sampling zone. Note: wounding and inoculation were performed on different plants.


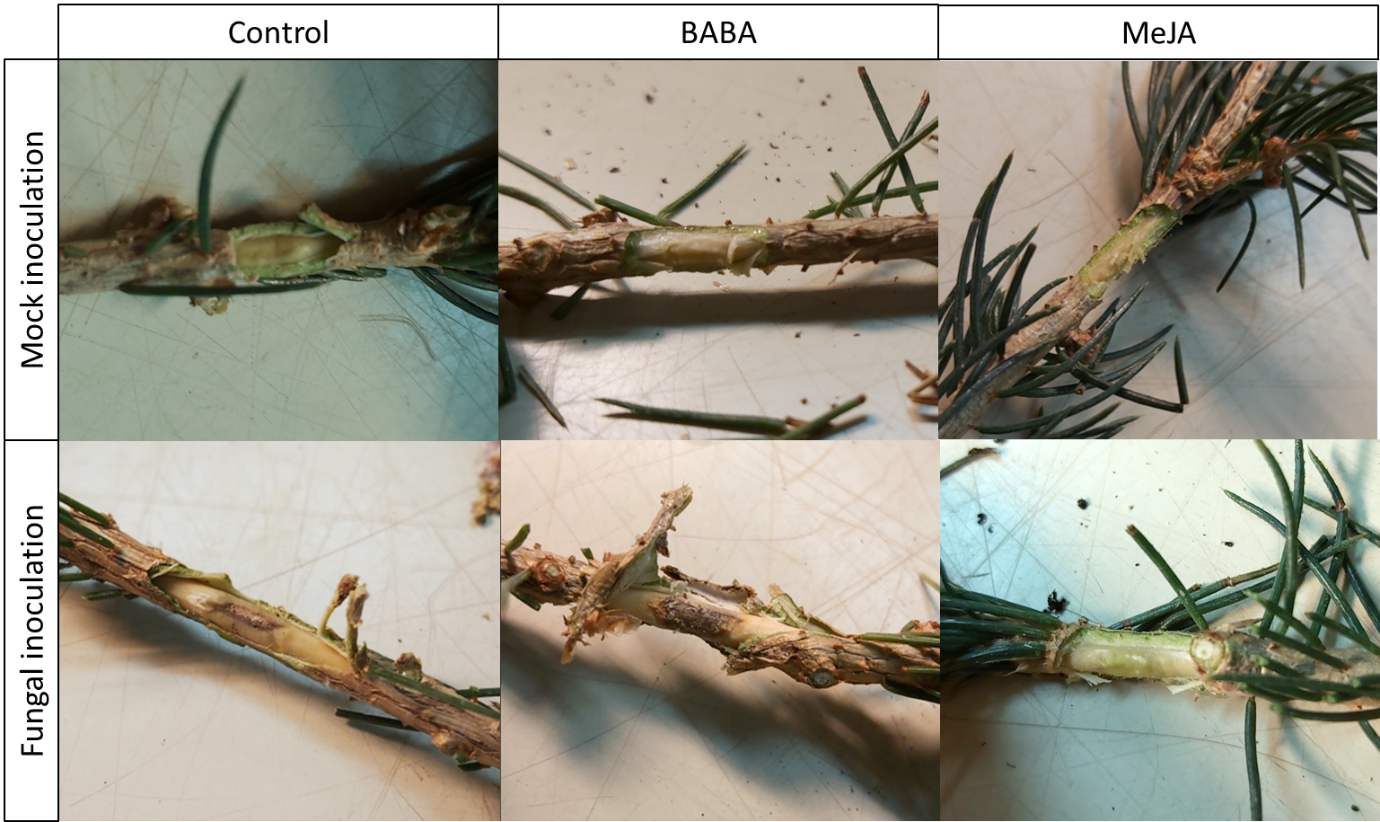


Supplementary Figure S2. Resistance phenotypes of 2-year-old Norway spruce plants inoculated with the bluestain fungus *Grosmannia penicillata* or mock controls 8 weeks before. Seedlings were treated with BABA or MeJA 4 weeks before inoculation or remained untreated as a control. Arrows point at the inoculation site. BABA: β-aminobutyric acid; MeJA: methyl jasmonate.

Supplementary Information.

Formulas for calculating (a) traumatic resin duct (TRD) coverage, (b) MeOH-insoluble tannin concentration, (c) MeOH-soluble tannin concentration, and (d) terpene concentration:

(a) TRD coverage = $\frac{Sum of TRD area at 5\times or 10\times magnification}{Xylem area at 2.5xmagnification}$ (µm^2^ µm^-2^ xylem area)

(b) Concentration = $\frac{\left( absorbance \right)-0.02112}{0.008*(sample weight)}$ (mg g^-1^ dry weight)

(c) Concentration = $\frac{4*(\left( absorbance \right)-0.02112)}{0.008*\left( sample weight \right)}$ (mg g^-1^ dry weight)

(d) Concentration = $\frac{TP/PD}{DW}$(µg g^-1^ dry weight)

TP is the area of a specified terpene compound peak in the chromatogram, PD is the area of the peak for the internal standard pentadecane, and DW is the sample dry weight.
